# Application of Inorganic Quantum Dots in Advanced Lithium–Sulfur Batteries

**DOI:** 10.1002/advs.202301355

**Published:** 2023-04-23

**Authors:** Zhuosen Wang, Haiyun Che, Wenqiang Lu, Yunfeng Chao, Liu Wang, Bingyu Liang, Jun Liu, Qun Xu, Xinwei Cui

**Affiliations:** ^1^ Henan Institute of Advanced Technology Zhengzhou University Zhengzhou 450001 P. R. China; ^2^ High & New Technology Research Center Henan Academy of Sciences Zhengzhou 450002 P. R. China; ^3^ Guangdong Provincial Key Laboratory of Advanced Energy Storage Materials School of Materials Science and Engineering South China University of Technology Guangzhou 510641 P. R. China

**Keywords:** catalysis efficiency, Li–S battery, polysulfides, quantum dots, shuttling effect

## Abstract

Lithium–sulfur (Li‐S) batteries have emerged as one of the most attractive alternatives for post‐lithium‐ion battery energy storage systems, owing to their ultrahigh theoretical energy density. However, the large‐scale application of Li–S batteries remains enormously problematic because of the poor cycling life and safety problems, induced by the low conductivity , severe shuttling effect, poor reaction kinetics, and lithium dendrite formation. In recent studies, catalytic techniques are reported to promote the commercial application of Li–S batteries. Compared with the conventional catalytic sites on host materials, quantum dots (QDs) with ultrafine particle size (<10 nm) can provide large accessible surface area and strong polarity to restrict the shuttling effect, excellent catalytic effect to enhance the kinetics of redox reactions, as well as abundant lithiophilic nucleation sites to regulate Li deposition. In this review, the intrinsic hurdles of S conversion and Li stripping/plating reactions are first summarized. More importantly, a comprehensive overview is provided of inorganic QDs, in improving the efficiency and stability of Li–S batteries, with the strategies including composition optimization, defect and morphological engineering, design of heterostructures, and so forth. Finally, the prospects and challenges of QDs in Li–S batteries are discussed.

## Graphical abstract

Quantum dots (QDs) with unique quantum confinement effects hold huge potential to boost the development of high‐energy‐density, Li–S batteries. In this review, a comprehensive overview is presented of the use of various QD‐based hybrid materials for advanced Li–S batteries, a guideline is provided of their structural designs with underlying mechanisms, and the prospects and challenges are discussed with suggested solutions.

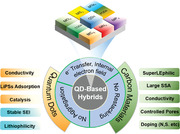

## 1 Introduction

The past decades have experienced fast industrial revolution in portable electronics, electric automobiles, smart grid, etc., because of the remarkable advancement in Li‐based battery technologies.^[^ ^1^, ^2^ ^]^ Li‐ion batteries (LIBs) are now the most widely‐used charge storage devices; nevertheless, after decades of research, the commercialized cathode and anode materials have almost reached their theoretical specific energy (420 Wh kg^−1^ based on insertion mechanisms).^[^ ^3^, ^4^, ^5^ ^]^ Furthermore, the rising crisis of global warming accelerates the necessity of replacing the gasoline‐powered vehicles with all‐electric vehicles (EVs), urging the development of a new electrochemical energy storage technology.^[^ ^6^, ^7^, ^8^ ^]^ In this regard, Li–S batteries have been considered as one of the most promising energy storage technologies among a variety of candidates, which demonstrate the following advantages. First and foremost, an ultrahigh theoretical energy density. With S as the cathode material and Li metal as the anode material, Li–S batteries possess the theoretical specific energies of 2600 Wh kg^−1^ and 2800 Wh L^−1^, which are over six times higher than those of LIBs. Moreover, sulfur is abundant, nontoxic, and cost‐effective, which appreciably raises our expectations for their applications in the energy storage sector.^[^ ^9^, ^10^, ^11^, ^12^, ^13^ ^]^ Thus, if the commercialization of the Li–S batteries has been realized, the cruising range of EVs will be substantially expanded, and the electrical mobility technologies will be greatly encouraged.

**Figure 1:**
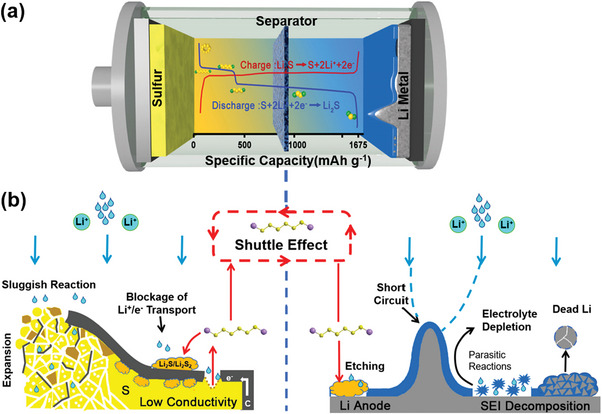
a) Typical Li–S cell configuration and the characteristic charging/discharging voltage profile. b) Schematic of the intertwined phenomena inducing the impaired reaction activity and the fast degradation of the sulfur cathode and Li metal anode during cycling.

In general, Li–S batteries use sulfur as the cathode and lithium metal as the anode, with both electrodes being electrically isolated and separated by a porous polymeric separator. The separator has selective permeability, allowing lithium ions to transport between the two electrodes through the electrolyte. The electrolyte is typically an organic liquid, which serves as an ion‐conduct medium that physically connects the two electrodes and assists the formation of solid–liquid interfaces.^[^ ^14^, ^15^, ^16^ ^]^ Except for these similarities, however, the reaction mechanism in a Li–S battery differs significantly from that in a LIB, in that it undergoes multi‐electron‐transfer conversion reactions, rather than insertion reactions.^[^ ^17^, ^18^ ^]^ **Figure** **1**a depicts the typical Li–S battery cell configuration. The relevant conversion reaction is readily seen in the image: 16Li + S_8_ ⇆ 8Li_2_S (assuming a complete reaction between the sulfur electrode and the Li metal electrode with the average potential E = 2.20 V vs Li/Li^+^).^[^ ^19^, ^20^, ^21^, ^22^ ^]^ Though the main chemical reaction looks simple, the detailed processes are quite sophisticated, which involve the formation of several soluble and insoluble lithium polysulfides (LiPSs) intermediates.^[^ ^23^, ^24^, ^25^, ^26^ ^]^ As also depicted in Figure 1a, during the discharge process, two different discharge stages can be recorded at 2.3 and 2.1 V, respectively. The first stage is related to the slow electrochemical conversion of sulfur molecules to long‐chain LiPSs (Li_2_S_8_→Li_2_S_6_→Li_2_S_4_, Li_2_S_n_, 4 ≤ n ≤ 8). Specifically, the solid phase S_8_ molecule is first converted into a soluble Li_2_S_8_, resulting in solid‐to‐liquid conversion. This procedure takes place on a high voltage platform. As the reaction proceeds, the Li_2_S_8_ continues to receive Li^+^ and transforms to polysulfide intermediates Li_2_S_n_ (4 ≤ n < 8) via a liquid‐to‐liquid conversion, with the corresponding voltage decreasing gradually due to the increased electrochemical polarization. It is worth noting that the first stage produces 25% (418 mAh g^−1^) of the predicted specific capacity of Li–S batteries. As the reaction progresses to the second stage, the long‐chain LiPSs are converted to the insoluble Li_2_S_2_/Li_2_S, and this step contributes a capacity of 1254 mAh g^−1^, which is 75% of the theoretical capacity. In the reversed process (charging process), on the other hand, two practical platforms overlap at 2.4∼2.5 V, which corresponds to the oxidation reactions from Li_2_S to LPSs, and further back to S.

It is the complicated charge‐discharge processes, and in particular, the solid–liquid–solid phase transitions during the conversion reactions in Li–S batteries, that cause severe commercialization and application hurdles (Figure 1b). To begin with, both sulfur and the fully discharged product of Li_2_S are electron insulator in nature, which increase the internal resistance of the battery, resulting in a poor electrical contact and slow redox reaction kinetic for the active material. Second, the density of Li_2_S is lower than that of sulfur (1.66 vs 2.03 g cm^−3^), which increases the volume of the cathode by 80% after the complete conversion from sulfur to Li_2_S.^[^ ^27^, ^28^, ^29^ ^]^ This considerable amount of volume change would lead to a number of safety issues, including the loss of the electrode integrity and the rapid capacity decay.^[^ ^30^, ^31^, ^32^, ^33^ ^]^ Third, the long‐chain LiPSs formed during charging and discharging processes are highly soluble in the electrolyte, which would diffuse into the electrolyte and transport to the anode side under the concentration gradient and the electric field.^[^ ^34^, ^35^ ^]^ This phenomenon, known as the “shuttling effect”, induces the continuous loss of the active materials during cycling, causing the impaired columbic efficiency and cycling stability.^[^ ^36^, ^37^, ^38^ ^]^ Finally, severe problems also exist at the Li‐metal anode side, including Li dendrite formation, large volume expansion/contraction, and continuous consumption of the electrolyte.^[^ ^39^, ^40^ ^]^ Although great progress has been achieved in rechargeable Li–S batteries in terms of the innovations on the effective electrolytes and unique cell configurations, their conversion efficiency and cycling stability still need to be improved for the practical application because of all the aforementioned barriers.^[^ ^41^, ^42^, ^43^ ^]^

Many efforts have been made over the last several decades to tackle these problems. Among these efforts, one of the most prominent strategies is to combine S with high conductivity matrix. It is generally known that the common carbon materials (such as carbon sphere, carbon nanotubes, graphene) can improve electrode conductivity and increase the S loading in the cathodes.^[^ ^44^, ^45^ ^]^ In addition, the increased specific surface area acts as a buffer against volume increase. However, because of the non‐polar nature of carbon‐based host materials, the inhibition of the shuttling effect is solely dependent on van der Waals interactions between the graphitic carbon and polysulfides. This physical absorption is too weak to stabilize polysulfides efficiently, and thus, it is necessary to increase the adsorption capacity of the carbon–S hybrid cathodes.^[^ ^46^, ^47^, ^48^ ^]^ In contrast to pure carbon‐based host materials, the hybrid materials that are functionalized by polar groups can provide abundance of polar sites to restrict the shuttling effect via chemical adsorption.^[^ ^49^, ^50^, ^51^ ^]^ As such, metal oxides,^[^ ^52^, ^53^, ^54^ ^]^ metal phosphide,^[^ ^55^, ^56^ ^]^ metal nitride,^[^ ^57^, ^58^ ^]^ metal carbide,^[^ ^59^, ^60^ ^]^ metal sulfides^[^ ^61^, ^62^ ^]^ metal–organic frameworks,^[^ ^63^, ^64^ ^]^ single atom catalysts (SACs),^[^ ^65^ ^]^ and other polar groups have been introduced as the S host and proven to effectively improve the adsorption of LiPSs on their surfaces. Moreover, the issue of the sluggish kinetics of the involved redox reactions must also be addressed. In recent years, catalysis concepts were developed to expedite electrochemical reactions in Li–S batteries, inspired by the techniques to improve the reaction kinetics for aqueous LiPSs.^[^ ^66^, ^67^, ^68^, ^69^ ^]^ Following this, an increasing number of studies on catalytic effects in Li–S batteries were published each year. It is generally understood that the catalytic effects could be substantially pronounced by reducing the size of the catalysts and increasing their exposure.^[^ ^70^, ^71^ ^]^ Furthermore, at the anode side, the lithiophobic nature of the carbon host can lead to non‐uniform lithium deposition, and thus the increase of lithiophilic nucleation centers through the addition of lithiophilic groups is highly desired. In a nutshell, the ideal hybrid materials should possess all the structural features shown above.

**Figure 2:**
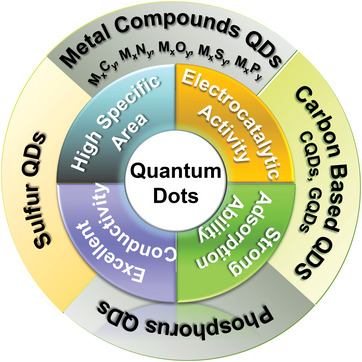
Schematic illustration of various types of quantum dots as well as their characteristics.

In this sense, quantum dot (QD)‐based nanocomposite hybrids have attracted considerable attention in energy conversion and energy storage devices. Quantum dots with particle size <10 nm have abundant active sites with quantum confinement effect, polar groups, high surface‐to‐volume ratios, and most are semiconductive.^[^ ^72^, ^73^, ^74^, ^75^ ^]^ As a result, QDs have apparent advantages over nanoparticles (>10 nm), such as good restriction of shuttling effect, excellent electrocatalytic activity, ability to buffer volume expansion, and the increased exposure of active surface area, as illustrated in **Figure** **2**. However, the quantum size effect would also cause the agglomeration of QDs because of their large surface energy. Fortunately, it is facile to uniformly integrate QDs with the conductive matrix (e.g., carbon matrix) to increase the overall conductivity of the sulfur cathode.^[^ ^76^, ^77^, ^78^ ^]^ The synergistic effects of QDs and the associated conductive carbon matrix offer the composite materials with exceptional physicochemical properties that may satisfy a wide range of criteria in Li–S batteries.

Numerous technologies have been employed to synthesize QD‐based hybrid materials with varied morphologies, such as spherical and tubular hollow structures, 2D‐nano sheets, and 3D network structures of hierarchical hybrids.^[^ ^79^, ^80^, ^81^, ^82^ ^]^ Previous reviews have summarized the application of QDs for energy conversion and storage in general.^[^ ^83^, ^84^, ^85^, ^86^ ^]^ Even in these reviews, they only focused on the carbon‐based QDs (carbon QDs, graphene QDs).^[^ ^87^, ^88^, ^89^ ^]^ In this paper, we extensively reviewed the most recent advances in the rational designs of QD‐based nanocomposites in Li–S batteries, with the emphasis on the optimal synthesis strategies as well as the structure‐performance correlations. First, the electrochemical reactions in Li–S batteries were described in detail, including the electrochemical reactions occurred at the sulfur cathode and at the Li anode throughout the charge/discharge processes. Various metal‐based and inorganic nonmetal QDs (Figure 2) loaded on different kinds of conductive carbon hosts were then reviewed, in the categories of their uses in the sulfur cathodes, Li anodes, and modified separators. Finally, we provided perspectives on the possible directions for the structural optimization and discussed the challenges about using QD‐based hybrids in Li–S batteries, with the suggested solutions to promote their commercialization in the future.

## 2 Electrochemical Reactions in Li–S Batteries

The electrochemical processes of Li–S batteries are quite intricate and involve a series of sequential conversion reactions.^[^ ^90^, ^91^, ^92^, ^93^ ^]^ Before fully discussing the roles of QDs in Li–S batteries, we will systematically describe the intrinsic hurdles occurred during the cathodic and anodic electrochemical reactions in Li–S batteries in this chapter (Figure 1b).

### 2.1 Conversion of S Species in Sulfur Cathode

A typical Li–S battery involves a series of sulfur‐based transitions from S_8_ to Li_2_S, as well as a solid–liquid–solid phase transformation during a discharge process.^[^ ^94^, ^95^, ^96^, ^97^, ^98^ ^]^ The discharge process can be divided into two stages as illustrated in the discharge curve (Figure 1a). To be more specific, it comprises a total of five sequential electrochemical reactions as shown below:

I) First, S_8_ (solid sulfur) acquires electrons and combines with Li^+^ to form a long chain of lithium polysulfides (LiPSs, Li_2_S_8_), which are highly soluble in the most commonly used ether‐based electrolytes. During this reaction, the active material of sulfur gains two electrons from the nearby conductive matrix, while Li^+^ diffuses from the electrolyte to sulfur. This step achieved a solid–liquid, two‐phase transition.^[^ ^99^ ^]^ The corresponding electrochemical reaction formula is shown as follows:

(1) $$3S_{8}+6e+6{\mathrm{Li}}^{+}↔3{\mathrm{Li}}_{2}S_{8}$$

II) Second, as the reaction proceeds, the liquidus Li_2_S_8_ transforms to the liquidus Li_2_S_n_ (4 ≤ n < 8), which are liquid–liquid, single‐phase transitions, demonstrated by the related formulas (2) and (3).^[^ ^100^ ^]^ The electrolyte with the dissolved long‐chain LPSs presents good fluidity. As a result, these LiPSs can easily diffuse to the Li anode side in the discharging step and then transport back and forth during the following cycling steps, generating the so‐called “shuttling effect”.^[^ ^101^ ^]^

(2) $$3{\mathrm{Li}}_{2}S_{8}+2e+2{\mathrm{Li}}^{+}↔4{\mathrm{Li}}_{2}S_{6}$$

(3) $$4{\mathrm{Li}}_{2}S_{6}+4e+4{\mathrm{Li}}^{+}↔6{\mathrm{Li}}_{2}S_{4}$$

Although the dissolution of LiPSs could improve the utilization ratio of solidus sulfur, the resulted shuttling effect significantly impairs the electrochemical performance of Li–S batteries, causing rapid capacity fading, low coulombic efficiency, and poor cycle stability. To prevent this detrimental effect, the phase transition rate of LiPSs should be accelerated so as to lower their concentration gradient in the electrolyte. In addition, the high affinity of the sulfur host to the LiPSs molecules is highly recommended.

III) Third, the dissolved Li_2_S_4_ are further converted to the insoluble Li_2_S_2_ via a liquid–solid transformation. Notably, because of its poor solubility, Li_2_S_2_ is readily saturated in the electrolyte and deposited on the surface of S host material, as shown in the corresponding formula (4).^[^ ^102^ ^]^ The energy barrier for the nucleation of the solidus Li_2_S_2_ causes a visible voltage decrease in this step. After deposition, the insulating Li_2_S_2_ would cover the S host materials, resulting in the blockage of the conductive channels within the active materials. As such, the polysulfides cannot get electrons from Li_2_S_2_, i.e., the polysulfides cannot be reduced at the surface of the existing Li_2_S_2_, resulting in capacity fading and incomplete sulfur consumption.

(4) $${\mathrm{Li}}_{2}S_{4}+2e+2{\mathrm{Li}}^{+}↔2{\mathrm{Li}}_{2}S_{2}$$

IV) Fourth, the solidus Li_2_S_2_ transforms to the solidus Li_2_S, resulting in a solid–solid, single‐phase transition, as illustrated in the formula (5). This is the most critical step because it contributes to a specific capacity of 836 mAh g^−1^, 50% of the theoretical capacity. However, unlike the previous steps, this step does not include the shuttling effect of LPSs and only involves the diffusion of Li^+^ in the solid phase. In addition, the electron transfer is also highly impeded because of the insulating Li_2_S_2_/Li_2_S configuration. Both aspects result in the sluggish reaction kinetics and high polarization. Thus, this step is the rate‐determining step of the entire discharging process.

(5) $${\mathrm{Li}}_{2}S_{2}+2e+2{\mathrm{Li}}^{+}↔2{\mathrm{Li}}_{2}S$$

Although the acceleration of homogeneous nucleation/growth of Li_2_S_2_/Li_2_S is considered to be critical to hasten this solid–solid, single‐phase transition,^[^ ^103^ ^]^ the mechanism has not been elucidated explicitly in the literature because the Li_2_S_2_ is not a stable phase and hardly to be observed experimentally.

### 2.2 Electrochemical Reactions at Lithium Metal Anode

Unlike ordinary LIBs, Li–S batteries always use lithium metal as the anode. Figure 1b also depicts a series of critical challenges of using Li metal as the anode, such as uncontrollable Li dendrite growth, unstable solid electrolyte interface (SEI) layers, and large volumetric and morphological changes, all of which lead to poor electrochemical performance and serious safety issues in Li–S batteries.^[^ ^104^, ^105^, ^106^, ^107^, ^108^ ^]^ Specifically, the unregulated deposition of Li causes Li dendrites to nucleate and develop fast. The separator may thus be pierced, resulting in an undesired internal short circuit of the batteries. In addition, the Li metal is known to be thermodynamically unstable because of its high Fermi energy level, and hence Li can react with the electrolyte and form thick SEI layers on its surface. This side reaction would cause the consumption of the Li metal and the electrolyte, an increase of battery internal resistance, and a decrease of coulombic efficiency. Moreover, since SEI layers are too fragile to buffer massive volume changes of the Li metal anode, the Li metal would experience endless volume expansion during the repeated plating and stripping procedures. Furthermore, the lithium metal is always attacked by the polysulfide anions shuttled within Li–S batteries, resulting in the corrosion and passivation of Li metal and a reduction in the utilization of sulfur. Because of these obstacles on the anode side, much effort has been taken on limiting Li dendrite growth.^[^ ^109^, ^110^, ^111^ ^]^

### 2.3 Fundamentals of Using QDs in Li–S Batteries

QDs are zero‐dimensional (0D) objects with electrons confined in all three dimensions. When reducing the size of the material down to its exciton Bohr radius (normally several tens to a few thousand atoms), the movement of electrons is restricted and quantum confinement effects are prominent.^[^ ^112^ ^]^ QDs are unique because of their size‐dependent electronic properties originated from the quantum‐confined structures. It is known that decreasing the size of QDs increases their effective band gap, and further, modifies their band structures from continuous band edges in the bulk materials into discrete electronic states as those in the atoms or molecules. Hence, QDs are often referred to as “artificial atoms”.^[^ ^113^ ^]^ As such, special properties such as multiple exciton generation, tunable band gaps, and the shift of photoluminescence, have been reported for QDs.^[^ ^114^ ^]^

In Li–S batteries, these quantum confinement effects are particularly interesting. On one hand, QDs can be tightly bonded (e.g., covalently bonded) onto the carbon conductive matrix, acting as the functional groups, so that they can affect the surface properties (e.g., electrolyte wettability, lithiophilicity, etc.) of the matrix while maintaining high electrical conductivity^[^ ^115^ ^]^ and high sulfur loading for the batteries. On the other, the highest occupied molecular orbital (HOMO) and the lowest unoccupied molecular orbital (LUMO) of the “artificial atoms” can be modulated according to the MOs of LiPSs molecules,^[^ ^116^ ^]^ facilitating their adsorption and the kinetics of their conversion reactions. More importantly, it projects an ideal scenario, the construction of multijunction of QDs, where each QD can be tuned for the adsorption and conversion of a particular LiPSs molecule, leading to the improved restriction of the shuttling effect and the enhanced catalytic effect along the whole electrochemical process. Although the multijunction design has not been realized for Li–S batteries, a significant research progress has been made in the past decade in solving the formidable problems illustrated in Figure 1b.

## 3 Design and Synthesis of QD‐Based Sulfur Cathodes

In this chapter, we present a comprehensive review of the creative designs of QD‐based hybrid materials that integrate active materials and conductive carbon supports. Various QDs are classified into two categories, metal‐based QDs, including metal carbides, nitrides, oxides/hydroxides, phosphides, sulfides, tellurides, and inorganic nonmetal QDs, including carbon, phosphorus, sulfur‐QDs. The summaries of the roles of QDs and the principles of designing QDs in the sulfur cathodes will also be discussed in this chapter.

### 3.1 General Synthesis Routes of QD‐Based Hybrid Materials

Generally, the fabrication of QDs can be classified into two groups of strategies, namely, top‐down and bottom‐up. The “top‐down” approaches refer to slicing, successive cutting, breaking, or etching the bulk structures into quantum pieces.^[^ ^117^ ^]^ The commonly‐used, “top‐down” techniques are lithography, ultrasonic exfoliation, reactive‐ion etching, ball milling, wet chemical etching, etc.^[^ ^85^ ^]^ These methods are beneficial for the large‐scale production; however, drawbacks exist, such as easy incorporation of impurities, imprecise control of size and shape, relatively weak bonding to the carbon matrix, and so forth.

The “bottom‐up” approaches are nowadays the most widely‐used methods in the synthesis of QD‐based hybrid materials for Li–S batteries. In these approaches, the QDs are in situ formed on the surface of the carbon matrix, by assembling small units (generally atomic or molecular) into the desired quantum structures. In a broad way, it can be further subdivided into wet‐chemical and vapor‐phase methods.^[^ ^118^ ^]^ The hydrothermal, sol‐gel, spray pyrolysis, and electrochemical methods are categorized into wet‐chemical methods;^[^ ^119^ ^]^ and chemical vapor deposition, atomic layer deposition, and sputtering can be categorized into vapor‐phase methods.^[^ ^120^ ^]^ In this review, most QDs were prepared by wet‐chemical, “bottom‐up” approaches, because of 1) the strong chemical bonding formed between the QDs and the carbon matrix, 2) precise control of the surface groups and electronic structures of QDs and the carbon matrix by adjusting the reaction conditions, and 3) the ease of incorporating high loadings of sulfur in the cathode. The typical wet‐chemical synthesis routes will be explicated in detail when introducing different types of QD‐based hybrid materials for Li–S batteries.

### 3.2 Metal Carbides QDs Based Nanocomposites

**Figure 3:**
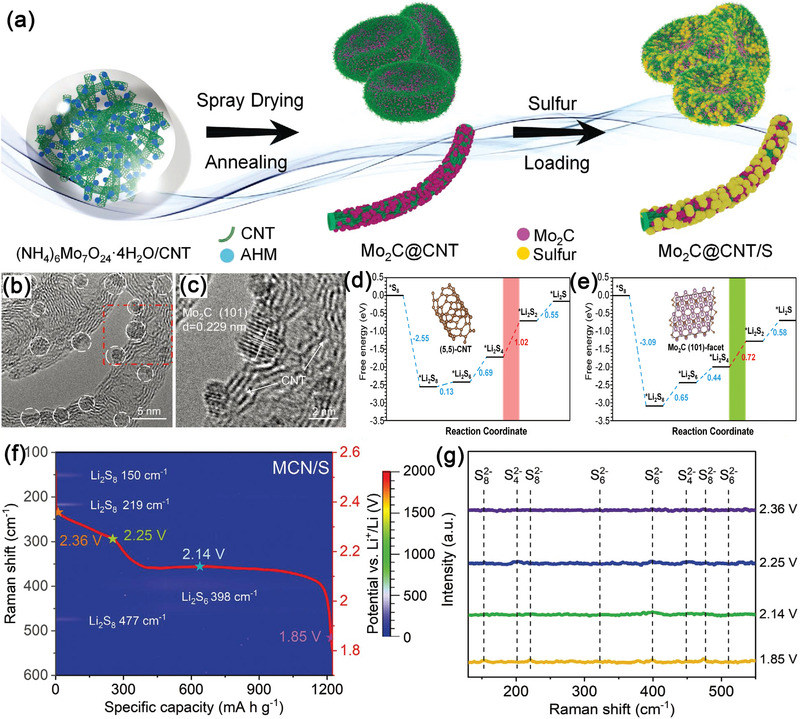
a) Schematic representation of the synthesis procedure of the MCN. b,c) HRTEM images of MCN. d,e) Energy profiles for the reduction of LiPSs on (5,5)‐CNT and Mo_2_C (101)‐facet, where the red dotted line represents the potential limiting step. f) In situ time‐resolved Raman images of the MCN/S. g) Selected Raman spectroscopy of the MCN/S cathode. Reproduced with permission.^[^ ^124^ ^]^ Copyright 2021, Wiley‐VCH.

Metallic Mo_2_C possesses high electrical conductivity (≈ 1.02 × 10^4^ S m^−1^), low cost, and strong polarity, and has been considered to be one of the most promising sulfur cathode possibilities.^[^ ^121^, ^122^, ^123^ ^]^ He and colleagues first published a Mo_2_C QDs‐decorated CNT network (MCN) through in situ spray‐drying technique, as illustrated in **Figure** **3**a–c.^[^ ^124^ ^]^ The as‐produced MCN electrode provided excellent cycling stability with rate capabilities of 1303.3 and 864.4 mAh g^−1^ at 0.2 and 1.0 C, respectively, and its corresponding degradation rate was only 0.019% after 1200 cycles under 1.0 C (**Table** **1**). Density functional theory (DFT) simulations and in situ Raman spectroscopy measurements were also employed to disclose the electrocatalytic mechanisms of MCN. As illustrated in Figure 3d,e, the rate‐determining step for the conversion of polysulfides was from *Li_2_S_4_ to *Li_2_S_2_. It was shown that the energy barrier of this rate‐determining step reduced from 1.02 eV for pristine CNTS to 0.72 eV for Mo_2_C QDs, implying the structural advantages of MCN in improving the kinetics of lithium sulfide reduction. Furthermore, in situ Raman spectroscopy measurements displayed in Figure 3f,g also revealed that MCN electrodes could efficiently restrict the shuttling effect of LiPSs, which can be ascribed to the good chemisorption ability of Mo_2_C QDs toward LiPSs.

**Table 1:** Summary of typical QD‐based sulfur cathodes

Cathode	Areal sulfur loading [mg cm^−2^]	Current Density [C]	E/S [µL mg^−1^]	Cycle number	Initial & retained discharge capacity [mAh g^−1^]	Ref.
Mo_2_C‐CNT	2.1‐2.5	1.0	15	1200	NG/691.1	ref. [124]
VNQD‐HG	1.3‐1.5	0.2	20	100	1200/1008	ref. [127]
VN‐H‐C	1.5	1.0	NG	500	856/603.4	ref. [128]
TiO_2_ QDs@MXene	1.5	2.0	NG	500	680/850	ref. [131]
TiO_2_‐QDs/N‐G	3.3	0.3	10	500	801/561	ref. [132]
OV–T_n_QDs@PCN	2.2	2.0	10	1000	750/660	ref. [133]
TiONQDs@C	NG	0.1	NG	200	906/869	ref. [134]
rGO@ZnO QDs	1.0	1.0	NG	400	998.8/918.8	ref. [135]
HPC@TOH	4.25	0.2	15	120	1076/1185	ref. [136]
NiFe_2_O_4_ QDs	4.7	0.1	4.26	500	910/500.5	ref. [137]
ZCO‐QDs@HCS	4.5	0.2	11	80	815.3/709.5	ref. [138]
MPQ@G	2.3	1.0	15	600	875.5/644.4	ref. [148]
CoNiP‐rGO	2.0	1.0	NG	600	NG/429	ref. [149]
CNT/CdS‐QDs‐30%	2.0‐2.2	0.5	25	150	1122.6/820.6	ref. [139]
CdS@NG‐CNT	1.0	1.0	NG	300	1102.1/756.1	ref. [140]
rGO‐MoS_2_ QDs	1.3‐1.5	1.0	NG	300	410/503	ref. [141]
ZnS QD@rGO	5.1	0.1	8	100	1011/800	ref. [142]
MTQ@3DG	2.4	1.0	15	600	842.4/711.7	ref. [150]
GQDs	1.0	0.5	NG	100	NG/1000	ref. [154]
GQD/Fe_2_O_3_@S@SnO_2_	2.6	0.5	NG	100	NG/1559	ref. [155]
GQDs	NG	0.1	NG	100	NG/344	ref. [156]
PEI‐CDots	6.6	4.8	15	400	694.4/500	ref. [157]
BPQDs	8	0.1	6.5	200	NG/550	ref. [163]
SQDs	1.0	0.5	NG	600	NG/766.4	ref. [167]

### 3.3 Metal Nitrides QDs Based Nanocomposites

**Figure 4:**
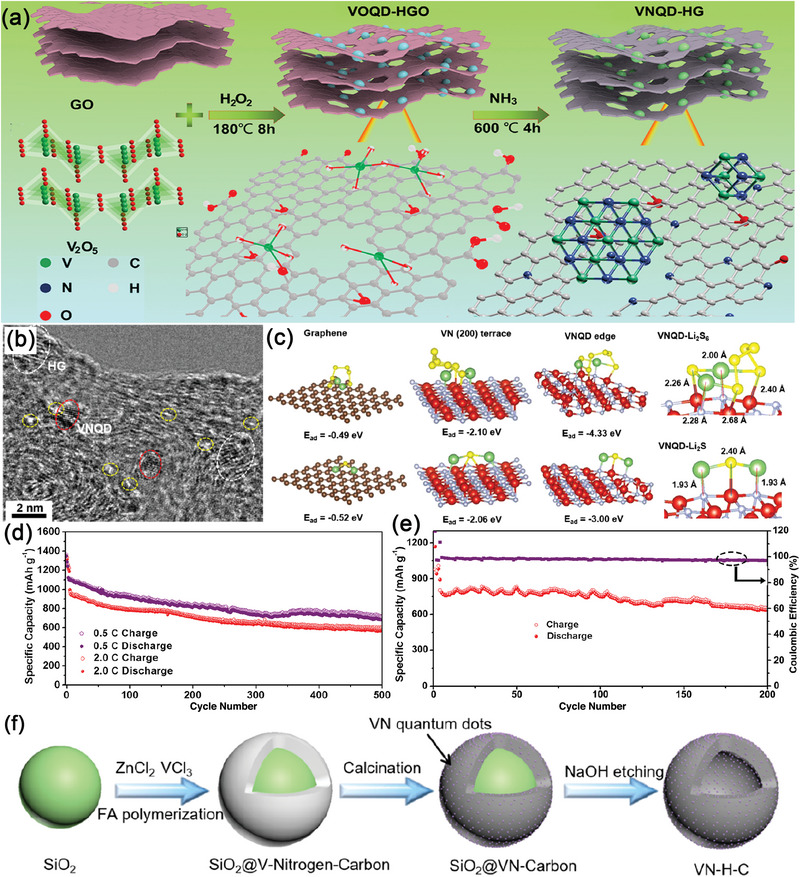
a) Schematic diagram of the synthesis process of VNQDs anchored onto nitrogen‐doped holey graphene matrix (VNQD‐HG). b) HRTEM image of VNQD‐HG. c) The adsorption energy of Li_2_S_6_ and Li_2_S on the surface of graphene, VN (200) and VNQD. d) Cycling stability of S/VNQD‐HG. e) Cycling stability of Li–S full batteries with limited Li excess of 200% with 10 µL mg^−1^ lean electrolyte. Reproduced with permission.^[^ ^127^ ^]^ Copyright 2021, American Association for the Advancement of Science. f) Schematic illustration of the synthetic process of the VN‐H‐C sample. Reproduced with permission.^[^ ^128^ ^]^ Copyright 2021, American Association for the Advancement of Science.

Compared with metal carbides, transition metal nitrides also have metallic characteristics, unique band gaps, outstanding catalytic properties, and high chemical stability, the QDs of which have lately emerged as the candidates for advanced Li–S batteries.^[^ ^125^, ^126^ ^]^ Yu and colleagues pioneered the introduction of VN QDs into the holey graphene matrix (VNQD‐HG) as the sulfur host (**Figure** **4**a,b).^[^ ^127^ ^]^ Owing to the abundant catalytic edge sites on VNQDs, the hybrid material possessed a high adsorption capacity and binding ability for Li_2_S_6_ and Li_2_S, and thus, improved the conversion kinetics of LiPSs, as shown by DFT (Figure 4c). Furthermore, the highly‐packed VNQD‐HG composites had a lot of out‐of‐plane and in‐plane nanopores, which could provide abundant electron and Li^+^ diffusion channels. As illustrated in Figure 4d, the VNQD‐HG electrode could achieve an outstanding electrochemical performance of 1320 mAh g^−1^ at the first cycle, with the corresponding capacities retention being as high as 99.95% after 500 cycles. Importantly, a full Li–S battery made of VNQD‐HG electrodes using 10 µL mg^−1^ lean electrolyte could also work for over 200 cycles without evident capacity decline (Figure 4e). In another study, VN QDs were introduced into the N‐doped mesoporous carbon shell (VN‐H‐C) as the host for the advanced S electrode.^[^ ^128^ ^]^ The VN‐H‐C were synthesized using a simple hydrothermal and carbonization technique (Figure 4f). One‐pot solvothermal method was first adopted to polymerize formamide along the SiO_2_ hard templates together with ZnCl_2_ and VCl_3_ at 180 °C, during which formamide connected together forming rich multiple N‐dentate ligands. The collected SiO_2_@V‐N‐C powders were then calcinated at 900 °C under Ar atmosphere, using the N‐containing groups as a nitrogen source for VN, which was followed by NaOH etching. The as‐prepared VN‐H‐C composite had proven to possess a highly porous hollow structure and excellent electrical conductivity. Additionally, due to the acceleration of interfacial charge transfer kinetics, the VN QDs could promote the nucleation and conversion of LiPSs. In this way, the VN‐H‐C sulfur cathodes provided exceptional electrochemical properties, and in particular, the long cycle stability as shown in Table 1.

### 3.4 Metal Oxides and Hydroxide‐QD‐Based Nanocomposites

**Figure 5:**
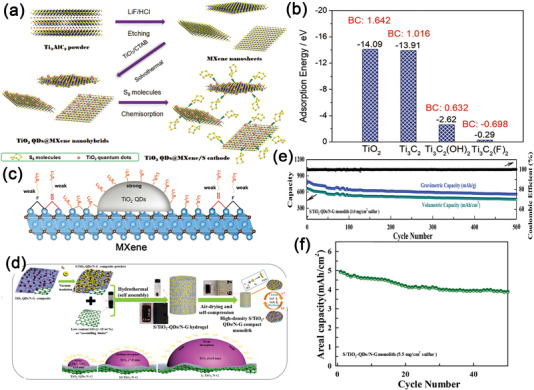
a) Schematic illustration of the fabrication of TiO_2_ QDs@MXene/S cathode. b) Adsorption energies of Li_2_S_4_ on different surfaces. c) Strategy for suppressing the shuttling effect of polysulfides by the growth of TiO_2_ QDs on MXene nanosheets. Reproduced with permission.^[^ ^131^ ^]^ Copyright 2018, Wiley‐VCH. d) Synthesis scheme of S/TiO_2_‐QDs/N‐G compact monolith and its electrochemical mechanism and size effect on capture ability of polysulfides. e) Long‐term cycling stability and its corresponding coulombic efficiency. f) Cycling performance at 0.1 A g^−1^. Reproduced with permission.^[^ ^132^ ^]^ Copyright 2020, Elsevier.

Despite that metal carbides and nitrides have unique metallic characteristics and catalytic properties, their chemical polarity to absorb LiPSs is a concern. In this sense, highly polar metal oxides such as TiO_2_ have attracted great attention to be used in the sulfur cathode.^[^ ^129^, ^130^ ^]^ In 2018, Liu group, for the first time, reported a hybrid material by growing TiO_2_ QDs on ultrathin MXene (Ti_3_C_2_Tx) nanosheets (TiO_2_ QDs@MXene), while cetyltrimethylammonium bromide (CTAB) was employed as a protective agent during the hydrothermal process, as illustrated in **Figure** **5**a.^[^ ^131^ ^]^ The TiO_2_ QDs were uniformly dispersed on the surface of MXene, preventing the restacking of MXene nanosheets. Furthermore, as shown in Figure 5b,c, TiO_2_ QDs have higher adsorption energy toward LiPSs than that of MXenes by DFT calculations. As a result, the produced TiO_2_ QDs@ MXene/S electrodes exhibited outstanding long‐term cyclability and rate capability (680 mAh g^−1^, 2 C, 500 cycles in Table 1).

Following this line, as shown in Figure 5d, Li and colleagues investigated the size effect of TiO_2_ nanoparticles on the electrochemical properties of Li–S batteries.^[^ ^132^ ^]^ In detail, Li et al. created a variety of sulfur hosts comprising different sizes of TiO_2_ nanoparticles (3.6, 7.8, 14.8 nm) loaded on nitrogen‐rich reduced graphene oxide (N‐G), which were distinguished as S/TiO_2_‐QDs/N‐G, S/M‐TiO_2_/N‐G, and S/L‐TiO_2_/N‐G electrodes. A series of electrochemical experiments and DFT calculations showed that the S host with the ultra‐small‐sized TiO_2_ QDs (TiO_2_‐QDs/N‐G) had the strongest adsorption energy and capture ability for LiPSs, which considerably improved capacity and cycling stability. The as‐synthesized S/TiO_2_‐QDs/N‐G presented a large volumetric capacity of 980 mAh cm^−3^ when the current density was set to 0.1 A g^−1^. In addition, after 500 cycles, it showed a slight degradation of 0.06% per cycle at 0.5 A g^−1^ (Figure 5e, Table 1). More importantly, as also illustrated in Figure 5f, this innovative hybrid electrode could achieve an actual capacity of 4.92 mAh cm^−2^ at 0.1 A g^−1^ with a low electrolyte/sulfur (E/S) ratio of 4.2 µL mg^−1^ and a high S loading of 5.5 mg cm^−2^.

**Figure 6:**
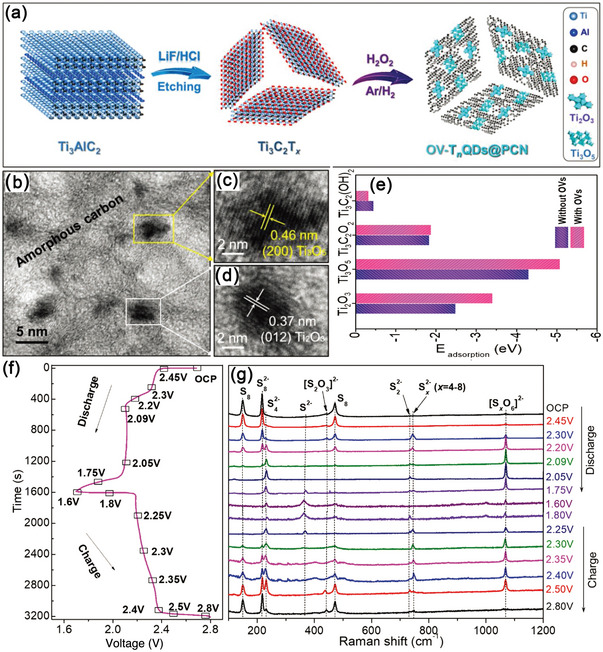
a) Synthesis process and morphological characterization of OV‐T_n_QDs@PCN. b–d) HRTEM images of OV‐T_n_QDs@PCN. e) Adsorption energies of Li_2_S_4_ with and without OVs. f) First discharging/charging profiles and g) corresponding in situ Raman spectra of OV‐T_n_QDs@PCN/S. Reproduced with permission.^[^ ^133^ ^]^ Copyright 2021, Wiley‐VCH.

Aside from the size effect, the defect engineering of TiO_2_ QDs had also been investigated to improve the reaction kinetics of LiPSs. Sun group, in 2021, presented a hybrid material with an oxygen‐vacancy‐rich (≈20%) Ti_n_O_2n−1_ QDs (OV–T_n_QDs, ≈3.3 nm)) anchored onto porous carbon nanosheets (PCN), OV–T_n_QDs@PCN (**Figure** **6**a–d).^[^ ^133^ ^]^ In the synthesis route, MXene (Ti_3_C_2_T_x_) nanosheets were used as the primary reactant, which acted both as the Ti and C sources for the preparation of OV‐T_n_QDs and as the 2D substrate to produce PCN. As confirmed by DFT and in situ Raman characterizations shown in Figure 6e–g, the introduction of oxygen vacancies boosted the immobilization and conversion of polysulfides by lowering the adsorption energy and shortening the bond lengths. The electrical conductivity should also be improved because of increased concentration of electrons and holes in OV‐T_n_QDs. As a result, the OV‐T_n_QDs@PCN/S exhibited exceptional rate capability of 672 mAh g^−1^ at 2 C (see Table 1) and excellent cycling stability (conservation rate of 88% after 1000 cycles at 2 C) with a sulfur loading of 2.2 mg cm^−2^. More importantly, the OV–T_n_QDs@PCN/S sulfur electrode also presented good Li^+^ storage of 736 mAh g^−1^ at 0.5 C over 500 cycles under a harsh condition of a low E/S ratio of 4.5 µL mg^−1^ and a high S loading of 4.8 mg cm^−2^.

**Figure 7:**
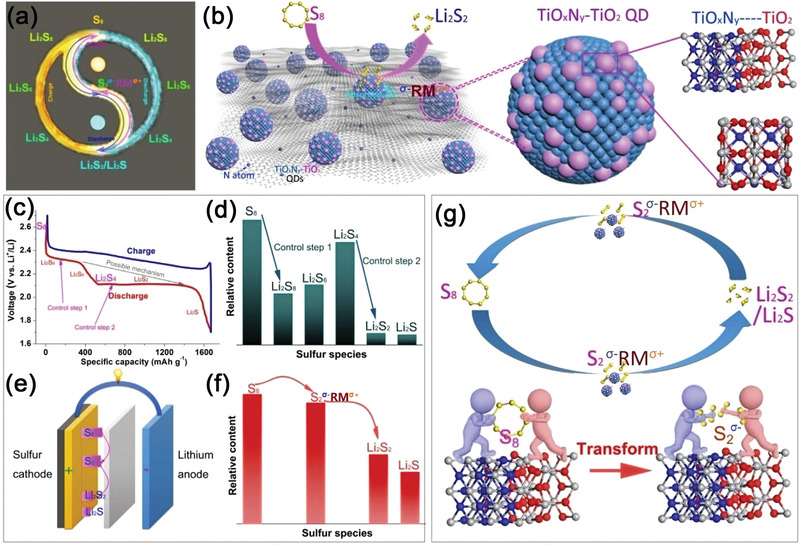
a) The possible conversion pathway of S species. b) Schematic diagram of TiO_x_N_y_‐TiO_2_ heterojunction. c) The charge/discharge curve of Li–S batteries. d) Relative contents of S species during the reaction process. e) Typical model of Li–S batteries. f) Relative contents of S species in a single control step. g) Schematic of TiO_x_N_y_‐TiO_2_ heterojunction convert S molecule into stable S_2_^*σ*−^ ‐RM^*σ*+^. Reproduced with permission.^[^ ^134^ ^]^ Copyright 2020, Elsevier.

In recent years, researchers have become increasingly interested in heterostructures. Kuang and co‐researchers designed and synthesized a novel sulfur host of TiONQDs@C composite as activator and adsorbent in sulfur cathodes (**Figure** **7**a).^[^ ^134^ ^]^ Figure 7b presented a schematic diagram of the structure of TiONQDs@C composite, in which large amount of TiONQDs were anchored on N‐doped carbon materials. The major component of the QDs was proven to be TiO_x_N_y_ (0 ≤ x ≤ y ≤ 1, x +y = 1) solid solution, with some micro‐regions occupied by smaller TiO_2_ islands, forming TiO_x_N_y_‐TiO_2_ heterostructures on the surface of TiONQDs. The authors then proposed that, by reducing the S_8_ molecules into small sulfur mediators (S_2_^*σ*−^Ti^*σ*+^‐TiON), this unique hybrid material could limit the formation of soluble LiPSs during the conversion reactions. The discharge curve in Figure 7c showed that there was only one platform present around ≈2.35 V and also one single reduction peak in the associated CV curve, indicating the simplified conversion reactions from S_8_ to S_2_^*σ*−^Ti^*σ*+^‐TiON and directly to Li_2_S_2_ molecules. Figure 7d–f depicted the related electrochemical reactions and the relative contents of species during the reaction. Benefiting from this new solid–solid reaction mechanism (Figure 7g), the TiONQDs@C/S cathode achieved a capacity of 869 mAh g^−1^ after 200 cycles at a current density of 0.1 C and a decay rate as low as 0.02% per cycle (Table 1). This article introduced a novel method to promote the kinetics and stability of Li–S batteries by avoiding the production of soluble LiPSs using heterostructured QDs.

Apart from the most popular TiO_2_ QDs, other metal oxide quantum dots, such as ZnO and tin oxide hydroxide QDs, were also used in Li–S batteries to act as the host for sulfur cathodes. Zhang et al. reported a ZnO quantum dot‐modified reduced graphene oxide (rGO@ZnO QDs).^[^ ^135^ ^]^ Since rGO@ZnO QDs have excellent catalytic effects, enhanced reaction kinetics, and large adsorption capacities for LiPSs, the hybrid electrode exhibited a distinguished rate capacity and cycling stability. Wang et al. synthesized tin oxide hydroxide quantum dots (TOH) loaded on a honeycomb porous carbon (HPC) matrix as multifunctional S hosts (HPC@TOH).^[^ ^136^ ^]^ The HPC had an excellent electrical conductivity and large specific surface area so that it could load a large amount of S and buffer the volume expansion during reactions. Thus, the HPC@TOH cathode could capture LiPSs physically and chemically, and promote their catalytic conversion. In this way, the HPC@TOH electrode realized a high initial capacity of 1342.95 mAh g^−1^ at 0.1 C in Table 1.

**Figure 8:**
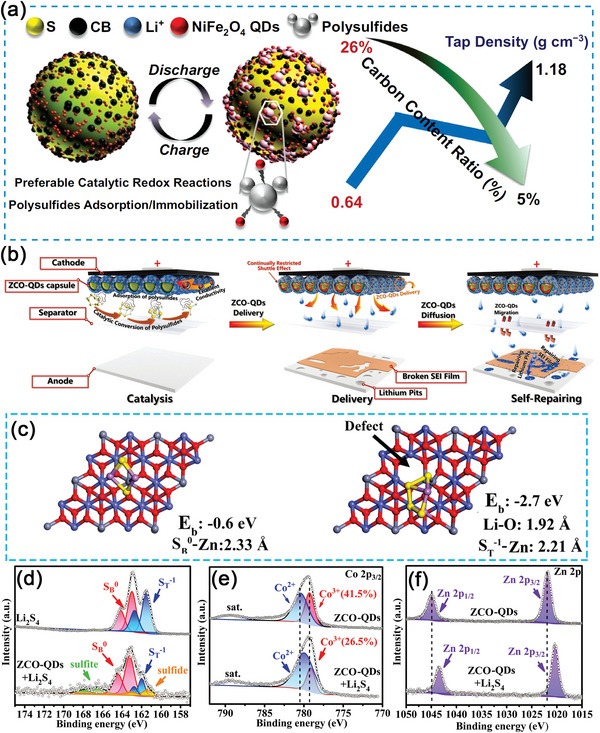
a) General diagram exhibiting configured devices of (−)Li//S@CB⊆QDs(+) and the reaction mechanism of NiFe_2_O_4_ QDs. Reproduced with permission.^[^ ^137^ ^]^ Copyright 2020, Springer. b) Schematic diagram of catalysis, delivery, and self‐repairing processes of ZCO‐QDs; c) Optimized atomic geometries of Li_2_S_4_ adsorbed on ZnCo_2_O_4_ (left) and defective ZnCo_2_O_4_ (right) with (100) crystal plane. d–f) XPS spectra of the pristine ZCO‐QDs and Li_2_S_4_/ZCO‐QDs. Reproduced with permission.^[^ ^138^ ^]^ Copyright 2021, Wiley‐VCH.

Compared to single metal oxides, bimetallic metal oxides can exhibit higher electrical conductivity and catalytic properties. Li et al. prepared multi‐functionalized NiFe_2_O_4_ QDs (excitation wavelength at 325 nm, emission wavelength at 568 nm) modified carbon materials (**Figure** **8**a).^[^ ^137^ ^]^ Due to their excellent electrical conductivity and plenty of utilization sites on their external surfaces, the implanted NiFe_2_O_4_ QDs could be used as “modular building blocks” in optimizing the components in sulfur cathodes for LiPSs utilization/catalysis. It was shown that, this unique sulfur host not only owned superb chemisorption interactions with soluble Li_2_S_n_ molecules and proper catalytic features facilitating polysulfide phase conversions, but could also cut down the carbon usage from 26% in traditional S/C cathodes to a low/commercial mass ratio of ≈5%. The reduction of carbon content could avoid the excess electrolyte consumption in Li–S batteries to guarantee their high‐energy‐density promise. In Figure 8b, Ni et al. reported another bimetallic metal oxide quantum dot of ZnCo_2_O_4_ QDs (ZCO‐QDs) that were embedded into the hollow carbon sphere (HCS) to constitute a ZCO‐QDs nanocapsule (ZCO‐QDs@HCS) as the multifunctional S host.^[^ ^138^ ^]^ Through DFT calculations (Figure 8c,d), in situ spectroscopic techniques, and various electrochemical tests (XPS, Figure 8e–g), the authors confirmed that the highly dispersed ZCO‐QDs@HCS effectively prevented the shuttling effect of LiPSs and promoted the conversion of polysulfides within the sulfur cathodes during reactions. In addition, hollow carbon nanospheres could greatly improve the conductivity of the whole cathode and promote the electrolyte infiltration and Li ion diffusion. Moreover, ZCO‐QDs could also act as a self‐repairing initiator to form a stable SEI layer on the Li anode side. Based on the advantages above, ZCO‐QDs@HCS exhibited an outstanding electrochemical performance in Li–S batteries even at a high sulfur loading of 4.5 mg cm^−2^.

### 3.5 Metal Sulfide QDs Based Nanocomposites

**Figure 9:**
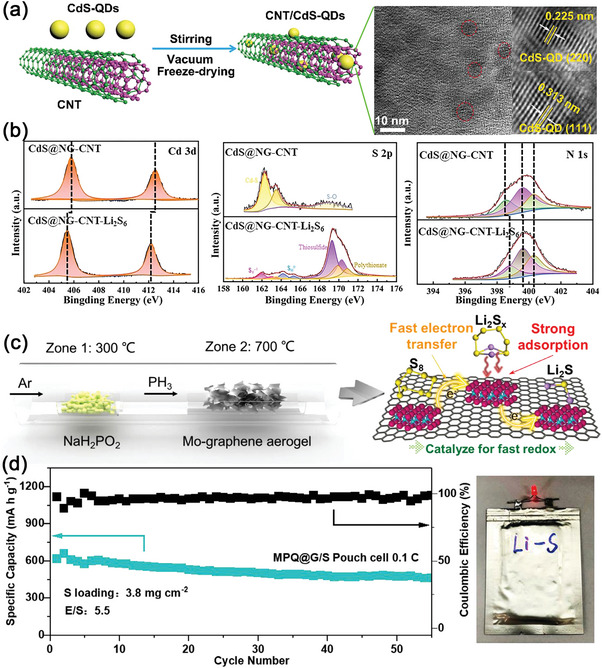
a) The diagram of the synthesis and structure of the CNT/CdS‐QDs composites. Reproduced with permission.^[^ ^139^ ^]^ Copyright 2019, Royal Society of Chemistry. b) Cd 3d, S 2p and N 1s XPS spectra of the CdS@NG‐CNT composite before and after Li_2_S_6_ adsorption. Reproduced with permission.^[^ ^140^ ^]^ Copyright 2021, Wiley‐VCH. c) Diagram of the synthesis of MPQ@G/S cathode. d) Cycling stability of the MPQ@G/S electrodes in pouch cells with an E/S ratio of 5.5 µL mg^−1^ and the photograph of MPQ@G/S pouch cell with a lighting LED device. Reproduced with permission.^[^ ^148^ ^]^ Copyright 2021, Elsevier.

In contrast to other metal‐based QDs, metal sulfide QDs are unique in terms of their strong sulfiphilic property to sulfur containing species. In 2019, Wei Han et al. synthesized CdS QDs via the solvothermal method by using S power and CoCl_2_ as precursors (**Figure** **9**a).^[^ ^139^ ^]^ Then, the CNT/CdS‐QDs composite materials were assembled using carbonyl multi‐walled CNTs as support. The effects of CdS‐QDs were also investigated by regulating its dosages in CNT frameworks, and the optimal dosage was determined to be 30% CNT/CdS‐QDs. The obtained CNT/CdS‐QDs composite loaded with sulfur exhibited an excellent electrochemical performance. At 0.5 C, the hybrid cathode achieved a capacity of 820.6 mAh g^−1^ after 150 cycles while the corresponding coulombic efficiency was >98.0% (Table 1). Furthermore, it exhibited an ultrahigh reversable capacity of 1053.9 mAh g^−1^ at 0.2 C. These findings and the corresponding experimental analyses indicated that the CNT/CdS‐QDs/S hybrid electrode displayed a unique configuration for the physical restriction of liquidous polysulfides and strong chemical coupling in facilitating charge transfer between CdS‐QDs and CNTs. Besides carbon nanotubes, Dongfang Niu et al. incorporated graphene oxide (GO) as a second support for CdS‐QDs and fabricated a 3D CdS@NG‐CNT composite material.^[^ ^140^ ^]^ The CdS@NG‐CNT/S hybrid electrode combined the advantages of low‐defect CNTs and graphene aerogel, which provided abundant macropores and mesopores for electrolyte infiltration and Li^+^ transportation. This unique 3D conductive network could also withstand large volume expansion of active materials. In Figure 9b, a low loading (5 wt.%) and uniform dispersion of CdS QDs on the 3D graphene and CNTs matrix could effectively confine LiPSs through chemisorption, promote their phase transformation, and accelerate the nucleation of Li_2_S to realize uniform Li_2_S deposition. Therefore, the 3D CdS@NG‐CNT/S electrode maintained a reversible capacity of 756.1 mAh g^−1^ at the 300th cycle at 0.5 C (Table 1).

Apart from CdS QDs, MoS_2_ QDs have also been introduced in sulfur cathodes. Hao Wei et al. reported a novel architecture with the nanosized MoS_2_ QDs decorated on a 3D structure of rGO as an S host.^[^ ^141^ ^]^ Due to large amount of sulfiphilic chemical bonds from MoS_2_ QDs, the composites exhibited outstanding chemical and physical absorption toward LiPSs. In 2020, Wei et al. also reported ZnS QDs and GO based hybrid material (ZnS QD@rGO) that could expedite polysulfide phase transformation.^[^ ^142^ ^]^ According to DFT calculations, the Fermi energy (E_F_) of graphene is −4.6 eV from the vacuum electron level, which is much higher than that of ZnS (E_F_ = −7.0 eV). Driven by this large potential energy difference, electrons spontaneously transported from graphene to ZnS at the interface, leading to strong interfacial polarization that effectively chemisorbs LiPSs for fast conversion reactions. This mechanism endowed the hybrid electrode of ZnS QD@rGO with an outstanding electrochemical performance (area capacity of 4.0 mAh cm^−2^, at 0.1 C). When the C rate increased to 1 C, the hybrid cathode exhibited a capacity retention of 91.2% after 300 cycles.

### 3.6 Metal Phosphide QDs Based Nanocomposites

Metal phosphides exhibit both excellent electrical conductivity and strong polarity to LiPSs, and therefore, are among the most popular host materials for S.^[^ ^143^, ^144^, ^145^ ^]^ Since MoP possesses the advantages of low band‐gap energy with higher electrical conductivity than other phosphides,^[^ ^146^, ^147^ ^]^ Chen et al. for the first time developed an MoP QDs loaded N, P co‐doped Graphene (MPQ@G) as a multifunctional sulfur host.^[^ ^148^ ^]^ As shown in Figure 9c, Mo‐graphene oxide aerogel was used as the precursor at downstream and NaH_2_PO_2_ was used as the phosphorus source at the upstream for the in situ phosphorization of Mo in the pyrolysis process. Within the hybrid structure, the N, P co‐doped graphene layers acted as skeletons to load the MoP QDs display, which could greatly improve electron/ion transport. Moreover, as a polar catalyst, the metallic MoP QDs could confine the shuttling effect of LiPSs by chemisorption and enhance the conversion rates of LiPSs. Therefore, the MPQ@G/S electrode exhibited a high capacity of 618.7 mAh g ^−1^ at 0.1 C in the first cycle and remained at 74.8% when the sulfur loading is 3.8 mg cm^−2^ and E/S is 5.5 (Figure 9d, Table 1).

Bimetallic metal phosphides have also been investigated. Zhou et al. synthesized a CoNiP QDs modified rGO and used it as a novel sulfur cathode in Li–S batteries.^[^ ^149^ ^]^ In this artful design, rGO nanosheets offered a highly conductive network and prevented the aggregation of CoNiP QDs. The corresponding DFT calculations and experimental results further proved that the doping of the second transition metal leaded to a smaller band gap, more superior surface activity, and higher structural stability. Therefore, the CoNiP‐rGO/S cathode exhibited a reversible discharging capacity of 429 mAh g^−1^ at 1.0 C after 600 cycles. Even when the sulfur loading increased to 6.0 mg cm^−2^, the CoNiP‐rGO/S cathode still exhibited a discharging capacity of 421.7 mAh g^−1^.

### 3.7 Metal Telluride QDs Based Nanocomposites

**Figure 10:**
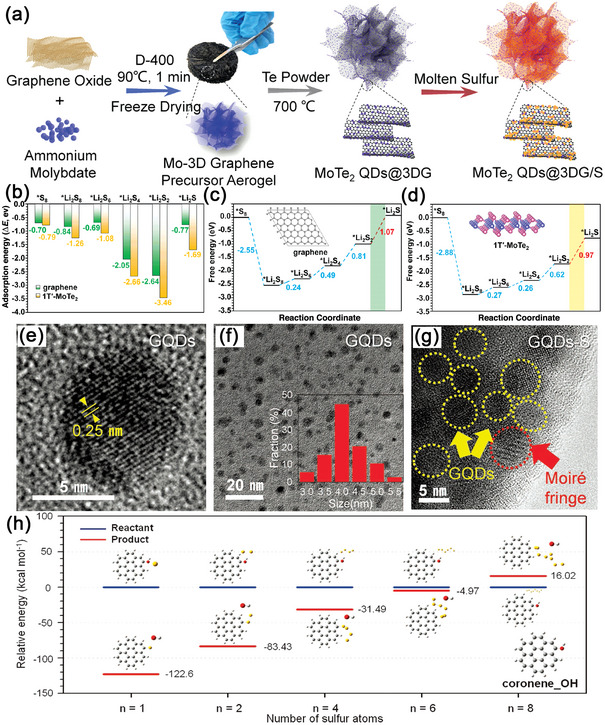
a) Scheme of the synthetic process for MTQ@3DG/S. b) Comparison of adsorption energies between Li_2_S_n_ and 1T’‐MoTe_2_ monolayer or graphene. c,d) Energy profiles for the reaction of Li_2_S_n_ on graphene and 1T’‐MoTe_2_ monolayer. Reproduced with permission.^[^ ^150^ ^]^ Copyright 2021, American Association for the Advancement of Science. e,f) HRTEM images of GQDs and g) GQDs‐S composites. h) The relative energies for the reactants and products in the binding of polysulfides to GQDs. Reproduced with permission.^[^ ^154^ ^]^ Copyright 2016, Nature Publishing Group.

In transition‐metal dichalcogenides (MoX_2,_ X = S, Se, Te), the electronic properties are significantly different for different anions and phases (2H, 1T, and 1T’). According to DFT calculations,^[^ ^150^ ^]^ as the atomic radius increases from S to Te, an increase in Mo‐X bond length can be noticed from Mo−S to Mo−Te in all phases of MoX_2_. In addition, 2H‐MoS_2_ possesses semiconductive properties with a direct band gap of 1.74 eV, which reduces to 1.52 eV for 2H‐MoSe_2_ and 1.15 eV for 2H‐MoTe_2_. However, 1T‐MoX_2_ and 1T’‐MoX_2_ phases present continuous band structures in the vicinity of the Fermi level, validating their metallic character and higher conductivity than 2H‐MoX_2_. In terms of structural stability, the 1T’ configurations are energetically more favorable than 1T ones. Based on these understandings, in 2021, Chen et al. reported 1T’‐MoTe_2_ QDs modified 3D‐graphene (MTQ@3DG) as a catalyst for advanced sulfur host (**Figure** **10**a).^[^ ^150^ ^]^ In addition to the high conductivity, the 1T’‐MoTe_2_ QDs also exhibited lower energy barrier for the nucleation of Li_2_S than that on the surface of pure graphene, which is thermodynamically favorable for the catalytic reactions during cycling (Figure 10b–d). In situ Raman analyses and electrochemical tests showed that the MTQ@3DG/S could successfully alleviate the shuttling effect of LiPSs through the endowed electrocatalytic activity. Thus, in Table 1, the MTQ@3DG exhibited a high initial specific capacity of 1310.1 mAh g^−1^ at 0.2 C, and a low fade rate of 0.026% per cycle after 600 cycles at 1.0 C. For practical applications, the authors constructed a pouch cell showing the capacity retention of 70.9% after 110 cycles at 0.1 C.

### 3.8 Carbon QDs Based Nanocomposites

**Figure 11:**
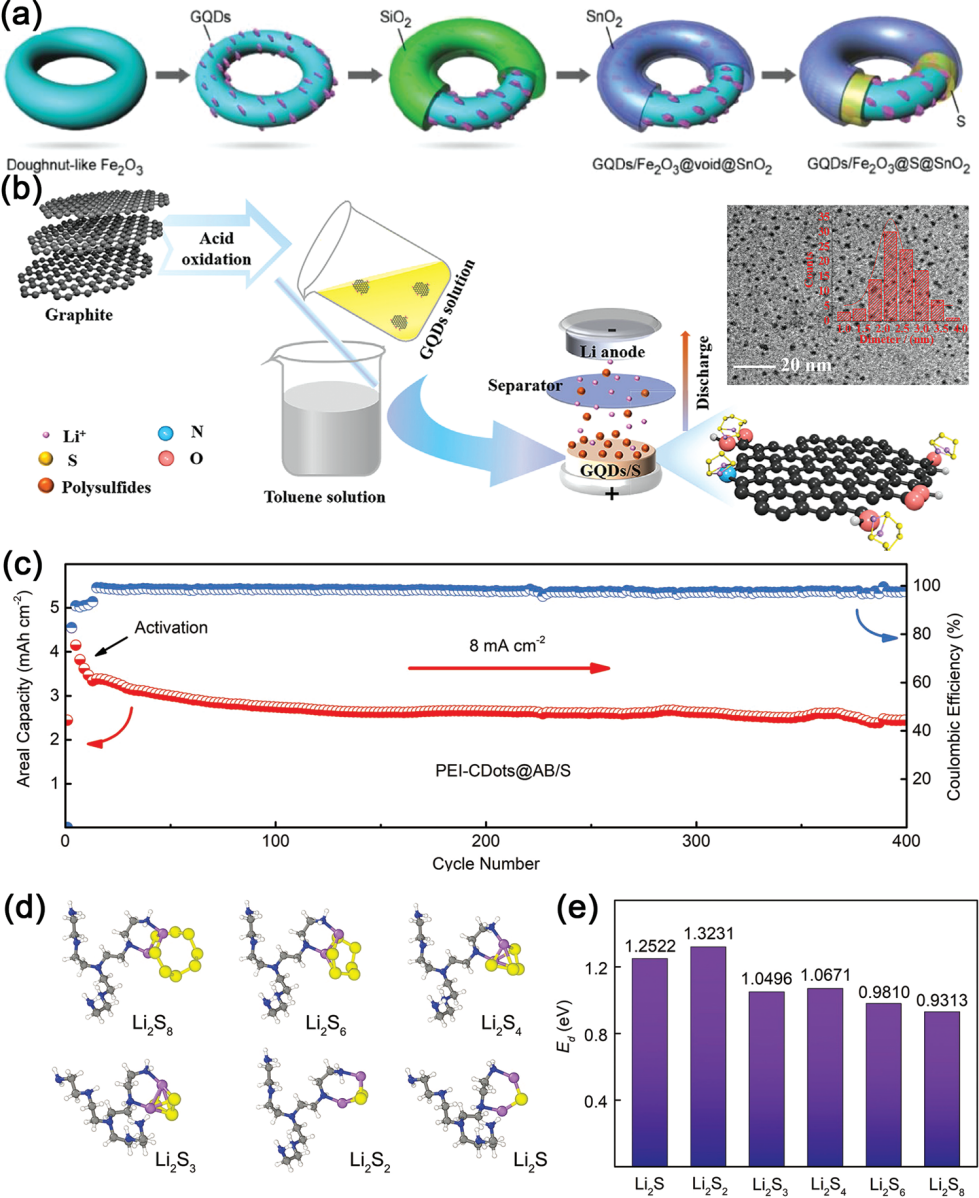
a) The diagram of the synthesis process of doughnutlike GQD/Fe_2_O_3_@S@SnO_2_ cathode. Reproduced with permission.^[^ ^155^ ^]^ Copyright 2021, American Association for the Advancement of Science. b) The diagram of the preparation of GQDs/S composite and TEM images. Reproduced with permission.^[^ ^156^ ^]^ Copyright 2021, Elsevier. c) Cycling stability of PEI‐CDots@AB/S cathode with the S loading of 6.6 mg. d) The calculated configurations of the LiPSs species on a reducible molecular structure of the PEI surface functional group. e) Binding energy between the PEI surface functional group and the LiPSs species. Reproduced with permission.^[^ ^157^ ^]^ Copyright 2018, Wiley‐VCH.

Because of high diversity in composition and properties, carbon quantum dots (CQDs) are emerging carbon materials with potential applications in various energy storage fields. CQDs are a class of spherical carbon nanoparticles (<10 nm in diameter) that include carbon nanodots (CNDs), graphene quantum dots (GQDs), and carbonized polymer dots (PDs). Among all types of CQDs, GQDs are 0D honeycomb sp^2^ carbon nanomaterials enriched with oxygen functional groups on their edges, whereby unique properties such as a non‐zero bandgap and luminescence on excitation have been reported.^[^ ^151^, ^152^, ^153^ ^]^ Furthermore, GQDs can uniformly cover the target material due to their small size, and thus, they are widely used in Li–S batteries. In 2016, Park et al. were the first to introduce GQDs as a sulfur host in Li–S batteries.^[^ ^154^ ^]^ In Figure 10e–g, GQDs showed an average particle size of ≈4 nm, which could enhance the structural integrity of a conventional micron‐sized sulfur–carbon electrode composite, forming a tightly packed structure. Importantly, due to the abundance of oxygen‐rich functional groups at the edge of GQDs, the C‐S bond was in situ formed during the reaction. Thus, GQDs could absorb the dissolved LiPSs and realize minimal loss of active materials (Figure 10h). Recently, Zhang et al. prepared a doughnut‐like core‐shell structure, GQDs modified ternary iron trioxide@sulfur@tin dioxide (Fe_2_O_3_@S@SnO_2_) hybrid material, for Li–S batteries (**Figure** **11**a).^[^ ^155^ ^]^ Both the Fe_2_O_3_ core and the SnO_2_ shell demonstrated strong binding for LiPSs. In addition, the GQDs loaded, doughnut‐like, yolk‐shell structure not only shortened the pathway for electron and Li^+^ transport, but also improved the utilization of S and specific capacity. Therefore, the GQD/Fe_2_O_3_@S@SnO_2_‐S cathode demonstrated a distinguished electrochemical performance, 923 mAh g^−1^ at 0.5 C after 100 cycles with the corresponding coulombic efficiency being close to 100% in Table 1.

Yang et al. treated flake graphite with strong acid and obtained GQDs with abundant oxygen‐containing functional groups and a few pyrrole‐N groups in chemisorbing LiPSs.^[^ ^156^ ^]^ The highly dispersed GQDs particles in the ethanol solution was then mixed vigorously with sulfur‐dissolved toluene solution before evaporation to be GQDs/S composite (Figure 11b). In this wet‐chemical synthesis method, GQDs served as nucleation sites, and the dissolved sulfur was reprecipitated around the GQDs, forming a dendritic interlaced network structure to prevent the aggregation of the sulfur, and further enhance the utilization of active substances. Thus, the chemical precipitated GQDs/S cathode delivered a high initial specific capacity of 1125 mAh g^−1^ at 0.1 C (in Table 1) and showed a low charge transfer resistance of 118 Ω, which was 3.5 times lower than that of GQDs/S_R_ cell prepared by physical melting method.

In addition to oxygen functional groups, other polar groups have also been attempted to modify CQDs. Polyethylenimine (PEI) is a branched polymer with plenty of amino groups. Xiong et al. reported PEI functionalized carbon dots (PEI‐CDots) and realized an excellent performance under high sulfur loadings and large C rates.^[^ ^157^ ^]^ When the PEI‐CDots@AB/S hybrid electrode was prepared with a high sulfur loading of 6.6 mg cm^−2^, the cathode exhibited a significant improvement area capacity of 3.3 mAh cm^−2^ at a current density of 8 mA cm^−2^ and the corresponding capacity decay rate was only 0.07% per cycle for 400 cycles (Figure 11c, Table 1). Through EIS, CV tests and DFT calculations, it was found that 0D PEI‐CDots increased Li^+^ migration around the solid‐electrolyte interface, and plenty of amine groups on the surface of CQDs provided abundant chemisorption sites to confine the shuttling effects of LiPSs (Figure 11d,e), endowing this PEI‐CDots modified sulfur cathode with a great potential for practical Li–S batteries.

Jun Lu et al., for the first time, reported an in situ encapsulation concept inspired by blood clotting for the protection of sulfur cathodes, which has been realized through the employment of N‐doped carbon dots (N‐CDs) as the electrolyte additive in Li–S batteries.^[^ ^158^ ^]^ In the presence of soluble LiPSs, N‐CDs became activated and stimulated the formation of a protective layer at the sulfur electrode–electrolyte interface to suppress the sulfur loss. It is reported that the formation of this LiPSs‐solidification layer is induced by the abundant N atoms and surface oxygen‐containing groups that strengthen the interaction of N‐CDs with LiPSs. Interestingly, the protective layer would disappear when dissolvable LiPSs are absent in the fully charged or discharged states. As a result, high sulfur utilization and long‐term cycling stability in Li–S batteries have been achieved using this strategy.

### 3.9 Phosphorus QDs Based Nanocomposites

**Figure 12:**
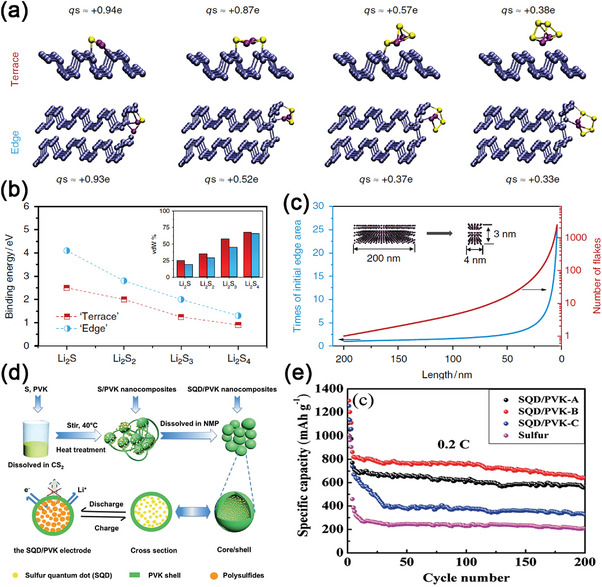
a) Snapshots of LiPSs adsorbed on terrace and edge sites of black phosphorus (BP) monolayer and nanoribbons, respectively. b) Binding energies of LiPSs adsorbed on terrace and edge sites of BP, the inset shows the fraction of van der Waals (vdW) contribution to the bond. c) The increase of exposed edge area and the number of flakes by downsizing a large BP flake to BPQDs. Reproduced with permission.^[^ ^163^ ^]^ Copyright 2018, Nature Publishing Group. d) Schematic illustration of the synthesis of the SQD/PVK composites and the Li^+^ diffusion process. e) Cycling performance of SQD/PVK nanocomposite and S electrode at the current density of 0.2 C. Reproduced with permission.^[^ ^167^ ^]^ Copyright 2015, Royal Society of Chemistry.

Black phosphorus (BP) is the most thermodynamically stable allotrope of phosphorus, with features of low resistivity, low density, fast Li‐ion diffusion constant, and high binding energies with sulfur.^[^ ^159^, ^160^ ^]^ These properties imply that BP should be able to form strong chemical bond with LiPSs and readily convert them to Li_2_S.^[^ ^161^, ^162^ ^]^ Inspired by this pioneer work, in 2018, Zhang et al. introduced BPQDs to the sulfur cathode for the first time and disclosed edge‐selective catalytic property of the BPQDs.^[^ ^163^ ^]^ There are two active sites in BPQDs for immobilizing LiPSs, the terrace sites and the edge sites (**Figure** **12**a). It was found that the binding energies of Li_2_S_n_ adsorbed at the edge of BP nanoribbons were significantly larger than those at terrace sites. In addition, the binding energy of Li_2_S_n_‐BPQD decreases when the size of the adsorbed molecule increases (Figure 12b). Therefore, the adsorption ability of LiPSs in BP can be largely increased by downsizing BP flakes to QDs (Figure 12c). After a small amount of BPQDs (2 wt.%) was introduced in a sulfur/porous carbon fiber cathode, no diffusion of LiPSs was observed during the conversion reactions, and thus, exhibited a distinguished electrochemical performance (784 mAh g^−1^ at 4 C and 0.027% capacity fade per cycle after 1000 cycles) in Li–S batteries in Table 1. Especially, when the sulfur loading increased to 8 mg cm^−2^, the cathode could still deliver a near 90% capacity retention after 200 cycles under lean electrolyte conditions.

### 3.10 Sulfur QDs Based Nanocomposites

Previous effort has been taken to infiltrate sulfur into oxides or conducting polymers, such as S@TiO_2_,^[^ ^164^ ^]^ S@PVP,^[^ ^165^ ^]^ S@PANI,^[^ ^166^ ^]^ etc., to confine the size of sulfur molecules. Following this line, decreasing the size of sulfur is an effective approach in improving the diffusion kinetics and simultaneously alleviating the volumetric expansion of sulfur. In this sense, sulfur QDs (SQDs) should be a good candidate in achieving high‐performing Li–S batteries. In 2015, Wang et al. developed core‐shell SQDs/PVK hybrid materials through a simple dissolution‐precipitation method (Figure 12d).^[^ ^167^ ^]^ SQDs (about 5 nm) with plenty of internal void spaces were encapsulated in the PVK shell. The internal void spaces accommodated volume expansion and prevented the dissolution of LiPSs. Furthermore, SQDs could shorten electron and Li^+^ diffusion distances and enhance the electrolyte wetting, and thereby improve the electrochemistry kinetics. As a result, SQDs/PVK‐S hybrid electrodes showed an initial discharge capacity of 861.6 mAh g^−1^ at 0.2 C, and after 200 cycles, it maintained 640.4 mAh g^−1^ with a capacity retention of 74% (Figure 12e). When the current density increased to 0.5 C, the cathode still exhibited a 488.6 mAh g^−1^ after 600 cycles. Although interesting, decreasing the size of sulfur sacrifices the sulfur loading that is strictly demanded for high energy density. Thus, the QD/micro‐sulfur composite design may be necessary for this direction in the future.

### 3.11 Conclusions and Further Discussion About QD‐Based Sulfur Cathodes

Based on the findings above, it can be concluded that the electrical conductivity, the restriction of the shuttling effect, and the catalytic ability are three critical aspects for the sulfur‐based cathodes in achieving excellent electrochemical performance and cycling stability. Compared to the catalytic additives with large sizes, QDs have size‐dependent, quantum‐confined structures and are very well suited as the sulfur host. First, QDs have diversified and adjustable electronic structures, which can be coupled with the conductive carbon matrix in improving the conductivity of the whole electrode and the utilization of S. Second, QDs can possess strong chemical polarity originated from polar metal‐anion covalent bonds or various functional groups on the surfaces and edges, which benefits to enhance the adsorption of LiPSs and reduce the shuttling effect. Thirdly, the quantum‐confined structures offer QDs with abundant active sites and atomic‐like density of states. As the size decreases, the quantum confinement improves the catalytic activity of QDs, effectively promoting the conversion of the LiPSs. Therefore, the QD‐based sulfur cathodes exhibit superior reaction kinetics as well as high utilization of sulfur with excellent capacities and cyclability.

Although various QDs decorated on carbon matrices have been attempted as the sulfur host in advanced Li–S batteries, any type of QDs has their intrinsic pros and cons. Metal‐carbide QDs (e.g., Mo_2_C QDs) and metal‐nitride QDs (e.g., VN QDs) have superior electrical and Li‐ion conductivity; however, their chemical polarity to absorb LiPSs is a concern. In contrast, metal‐oxide QDs (e.g., TiO_2_ QDs) have high polarity toward LiPSs because of the large electronegativity difference between metallic elements and O atoms; however, their band gap is relatively large, which will be expanded further by the quantum‐confined structures. Strategies, such as defect engineering (e.g., inclusion of vacancies and hetero‐atom doping) and bimetallic metal oxides, have been attempted to overcome these shortcomings with promising performance reported. It is suggested that the heterostructures combining metal‐carbide or metal‐nitride QDs with the optimized metal‐oxide QDs is an effective method to take full advantages of QDs. In terms of transition‐metal dichalcogenides (MoX_2,_ X = S, Se, Te), MoX_2_ QDs are unique in terms of their strong sulfiphilic property to sulfur containing species; however, their electronic properties are highly dependent on the dichalcogenide anions and phases (2H, 1T, and 1T’). Thus, continuous phase evolution of MoX_2_ QDs during reactions significantly deteriorates their cycling performance. In addition, some MoX_2_ QDs have been found to be chemically unstable in ether‐based organic electrolytes in Li–S batteries.^[^ ^150^ ^]^ CQDs have high diversity in compositions and properties because of various types of abundant functional groups. BP QDs have features of high conductivity and high binding energies with LiPSs because of the extensive exposure of highly active edge atoms. However, the structural instability of the functional groups on CQDs and reconstruction of the edge structures on BP QDs would cause the relatively fast deactivation of QDs. As also discussed above, SQDs sacrifice high sulfur loadings for high energy density. Therefore, among effective strategies attempted before, such as composition optimization, defect and morphological engineering, design of heterostructures, etc., it is believed that the integrated design of heterostructures between different QDs and between QDs and the carbon matrix, in particular the multijunction of QDs on carbon, should acquire special research attention for the sustainable improvement of the efficiency and stability of practical Li–S batteries in the future.

## 4 Design and Synthesis of QD‐Based Composites for Li Metal Anodes

It is well known that Li metal plays a key role in realizing high cycling life and superior property of Li–S batteries. It is described that Li dendrites could penetrate the separator and even touch the positive electrode, causing the short circuit and safety hazard.^[^ ^168^, ^169^, ^170^, ^171^ ^]^ More importantly, the generation of Li dendrites would require the formation of thick SEI films and consistently induce side reactions, leading to excessive consumption of the electrolyte and continuous reduction of coulombic efficiency. As a result, it is very necessary to control the deposition of Li and prevent the formation of dendrites. Because of plenty of active sites, unique electronic structures, and ultrafine particle size, QDs and their hybrid materials are regarded as one of the most promising materials in stabilizing Li metal anodes. A variety of approaches have been reported, such as the use of QDs as electrolyte additives and the construction of QD‐based protective layers for the Li metal.

### 4.1 Carbon QDs for Li Metal Anode

**Figure 13:**
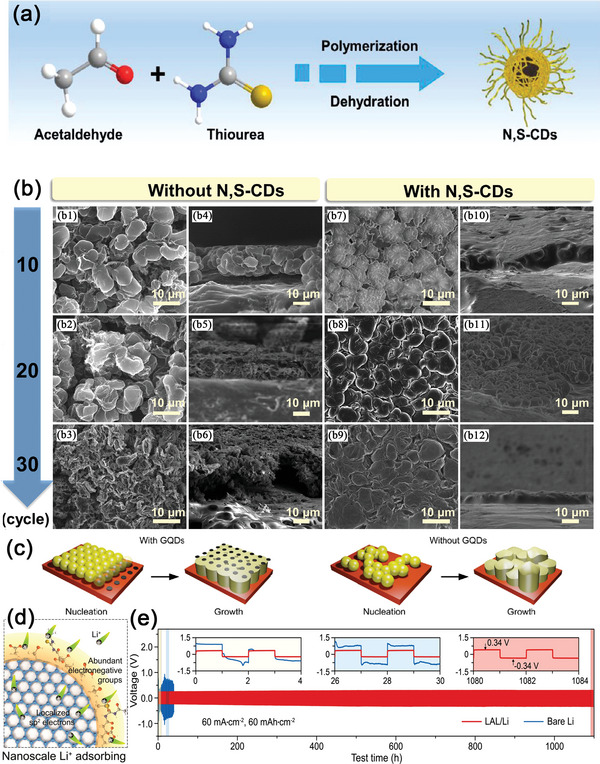
a) Schematic depicting the mechanism for the formation of N, S‐CDs. b) The SEM image of Li^+^ ion deposition in the Li|Li symmetric cell at 1.0 mA cm^−2^ with an areal capacity of 1.0 mAh cm^−2^. Reproduced with permission.^[^ ^172^ ^]^ Copyright 2021, Elsevier. c) Illustration of the GQDs regulated deposition processes. Reproduced with permission.^[^ ^174^ ^]^ Copyright 2016, Royal Society of Chemistry. d) The design rationale of the graphene quantum dot building blocks for the LAL. e) Galvanostatic cycling profiles of symmetric cells with the LAL/Li electrode or bare Li electrode. Reproduced with permission.^[^ ^175^ ^]^ Copyright 2021, Wiley‐VCH.

According to previous discussion, the homogeneous deposition of Li^+^ plays a key role in preventing Li‐dendrite formation. As shown in **Figure** **13**a, Hou and co‐workers have reported the use of N, S‐codoped CQD as an effective electrolyte additive to regulate Li^+^ deposition in an advanced Li–S battery.^[^ ^172^ ^]^ N, S‐codoped CQDs presented excellent dispersity in the electrolyte solution. When this modified electrolyte was used, N, S‐codoped CQDs could be uniformly deposited together with Li^+^ ion on the surface of the anode, restricting the formation of Li dendrites. As a control, when the untreated electrolyte was used, Li^+^ ions exhibited a random deposit during the charging process. Through the DFT calculations, the authors have demonstrated that both N and S atoms possess excellent adsorption ability toward Li^+^, and thus N, S‐codoped CQDs have plenty of lithiophilic sites so that Li^+^ ions can be adsorbed onto the CQDs and homogenously deposited thereafter. This phenomenon has also been recorded by the images of confocal laser scanning microscope shown in Figure 13b. As a result, with the present of N, S‐codoped CQDs in the electrolyte, the Li|Li symmetric cell delivered a superior stable cycle life 1200 h (600 cycles) at 1.0 mA cm^−2^ with an areal capacity of 1.0 mAh cm^−2^, and a significantly improve the capacity from 491 to ≈600 mAh g^−1^ after 200 cycles at 1.0 C was also realized in the Li–S full battery.

Different from typically spherical CQDs, GQDs with 1–3 graphene layers are anisotropic with 2D morphology.^[^ ^173^ ^]^ Thus, GQDs possess stronger quantum confinement effect and edge effect than those in CQDs in regulating Li^+^ deposition. In Figure 13c, GQDs were introduced into the electrolyte by Xiong's group.^[^ ^174^ ^]^ Polyethylene oxide (PEO) was also used as a protective agent to provide the gel‐like framework in avoiding the precipitation of GQDs. In this way, the GQDs in the gel electrolyte could control the Li^+^ distribution at the electrochemical interface and prevent the formation of Li dendrites. More importantly, through the SEM and in situ Raman tests, the authors analyzed the nucleation and growth process of metallic Li deposit in the Li‐copper asymmetry cell. It was found that the prepared GQDs acted as well‐distributed seeds for facilitating the homogenous nucleation of Li metal (Figure 13c). As a result, Li–S batteries containing the GQD‐modified gel electrolyte could realize high Coulombic efficiencies even under a high sulfur loading. The optimized Li–S batteries delivered an initial capacity of 2.3 mAh cm^−2^ at 0.4 C with the Coulombic efficiency higher than 99% at a high sulfur loading of 4 mg cm^−2^.

In addition to be used as the additive in the electrolyte, GQDs were also tried to be deposited directly onto the Li‐metal surface and acted as a protective film for the anode. Peng and co‐workers have synthesized a new type of GQDs, which were tailored by various polar functional groups containing —OH, —NH_2_, C=O/C=N, C=C, and C=S bonds. After coated onto the surface of Li metal, the grafted polar functional groups on GQDs formed a Li^+^ adsorbing layer (LAL). This LAL layer could selectively adsorb the Li^+^‐ion flux at the electrolyte–anode interface, and make Li^+^ ions evenly distributed and homogenously deposited during the charging process, as shown in Figure 13d.^[^ ^175^ ^]^ Furthermore, the LAL‐modified GQDs could also increase the local Li^+^ concentration at the interface preferentially, so as to restrict any depletion of Li^+^ ions at high current densities. Benefiting from these advantages, the modified Li‐metal anodes realized a long‐term reversibility for Li plating/stripping, over 1000 h at a high current density of 60 mA cm^−2^, as shown in Figure 13e.

### 4.2 Metal Compound QDs for Li Metal Anode

**Figure 14:**
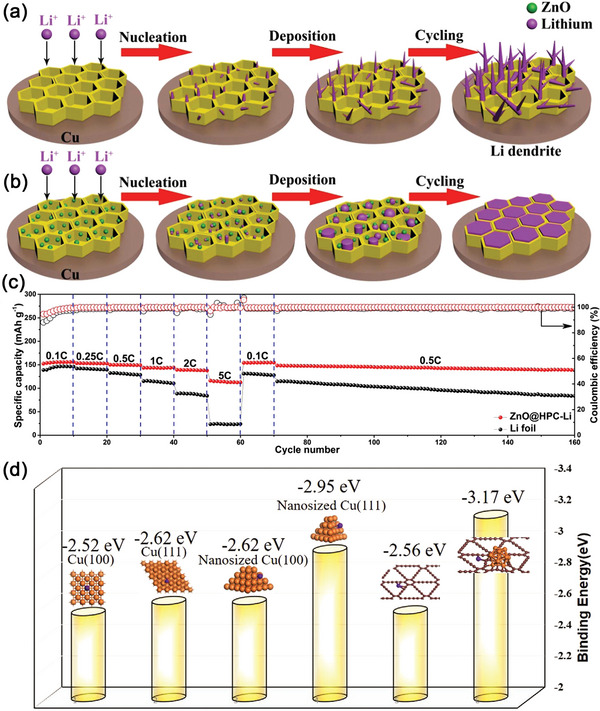
a,b) Schematic diagrams for the comparison of Li deposition within HPC scaffold a) with and b) without the decoration of ZnO nanoparticles. c) Rate capability of LCO/ZnO@HPC‐Li and LCO/Li cells. Reproduced with permission.^[^ ^177^ ^]^ Copyright 2017, Elsevier. d) The corresponding binding energy of the Li atoms on different sites of the CuQD@GDY. Reproduced with permission.^[^ ^178^ ^]^ Copyright 2020, Wiley‐VCH.

Besides CQDs and GQDs, metal compound‐based QDs were also used to modify the Li metal anodes. In early 2012, Park and co‐researchers have prepared ZnO QDs with the size of ≈3.5 nm, which uniformly covered the hierarchically porous carbon supports made from the pyrolysis of the metal–organic framework (MOF‐5).^[^ ^176^ ^]^ The structural advantages of this hybrid material had been recommended for energy storage applications. Inspired by this, in 2017, Tao's group fabricated ZnO QDs distributed on a bamboo‐derived, 3D hierarchical porous carbon (HPC) for the stabilization of Li metal anodes.^[^ ^177^ ^]^ As shown in **Figure** **14**a, without the modification by ZnO QDs, the 3D HPC could easily induce Li‐dendrite formation because of the lithiophobic nature of carbonaceous materials. However, after the incorporation of ZnO QDs with the size ≈5 nm (Figure 14b), the Li^+^ deposition could be highly regulated within the honeycomb‐shaped carbon skeletons. As a result, the ZnO@HPC electrodes could achieve an outstanding cycling stability with high Coulombic efficiencies (Figure 14c). Moreover, the authors also systematically investigated the growth behavior of Li dendrites within the ZnO QD‐modified 3D HPC network. It has been confirmed that the anchored ZnO QDs could effectively reduce the overpotential of Li plating and stripping because of the redox reactions between Li^+^ and ZnO. Thus, Li preferred to nucleate on the surface of ZnO QDs rather than directly deposit on HPC. More importantly, as the reaction continued, Li would react further with the reduced Zn and generate a highly conductive alloy of LiZn. This process not only produced lots of pores within ZnO QDs, but also increased the lithiophilicity of the whole electrodes, both of which would facilitate the uniform deposition of Li^+^ on the anode.

Li and co‐workers in situ generated the Cu QDs during the synthesis of graphdiyne (GDY), forming CuQDs@GDY hybrid materials for stabilizing the Li‐metal anode.^[^ ^178^ ^]^ The authors used polycrystalline Cu nanowires (CuNWs) as both the support and the catalyst to grow GDY nanosheets. They found that the grain boundary in CuNWs possessed high catalytic activity for the generation of intermediate Cu‐acetylide, and thus, polycrystalline CuNWs would be pulverized into Cu QDs during the growth of GDY nanosheets. The obtained CuQDs@GDY delivered an excellent cycling performance for Li‐metal batteries (remains about 73% after 500 cycles, much higher than that of the Li metal 38%). DFT calculations showed that Li atoms could have higher binding energy with CuQD@GDY than those with pure Cu or pure GDY (Figure 14d). This result revealed that the improved stability of Li metals was originated from the enhanced lithiophilicity of the hybrid microstructure. The spontaneous production of CuQDs@GDY was also suggested to be a common strategy for the synthesis of QD‐based hybrid materials, which may be wildly utilized in energy storage as well as many other energy‐related fields, such as water splitting and fuel cells.

### 4.3 Other QDs for Li‐Metal Anodes

Cui's group have fabricated a novel framework of silicon (Si) nanoparticles embedded reduced graphene oxide (SirGO) as the hybrid host of Li metal.^[^ ^179^ ^]^ The embedded Si nanoparticles have been proven to be lithiophilic and could act as the nucleation seeds for the uniform deposition of Li. Based on this, Zhang's group have further prepared a prelithiated Si QDs@rGO thin film anode, where Li was electrodeposited between restacked Si QDs@rGO nanosheets before cycling.^[^ ^180^ ^]^ In addition to the improved lithiophilicity, it was claimed that Si QDs with ultrafine size could also maintain the parallel stacking of rGO layers and confine the Li^+^ deposition through the thickness direction. As result, the sandwiched structure realized a uniform Li plating and facilitated the generation of stable SEI layer, both of which could substantially enhance the cycle stability of lithiated Si@rGO when used as the Li‐metal anode.

Based on previous discussion, much effort has been taken on limiting Li dendrite growth through the optimization of the electrolyte composition as well as the increase of the lithiophilic groups on Li hosts. It is suggested that the structural designs that can endow QDs with improved lithiophilicity and enhanced dispersity within the conductive carbon matrices are highly desired. In addition, the use of QDs in stabilizing SEI film and in forming artificial SEI films are also effective strategies for Li anode protection, which may require substantial research effort in the future.

## 5 Design and Synthesis of QD‐Based Interlayers and Separators

The aforementioned methods mainly focus on confining LiPSs and Li^+^ distribution within the electrode. Apart from interior modification strategies, exterior restriction of the shuttling effect as well as lithium dendrite formation by a modified separator is also a critical and attractive strategy, since it provides extra protection along the diffusion pathways of polysulfide anions and Li cations. The modified separators used in Li–S batteries have the following distinctive advantages: i) This is a “two‐in‐one” strategy, which can not only restrain the shuttling of LiPSs originated from the S cathode side, but also prevent the uneven deposition of Li^+^ on the anode side. ii) It can be coupled with interior modification strategies to provide multi‐level and synergistic protection to overcome the formidable problems in Li–S batteries. iii) Only a small amount of coating material is required for the protection, assuring high sulfur loading for high energy density. iv) The coating methods are normally facile and cost‐effective, which is an essential asset in mass production. A number of coating materials for separators have been investigated, including carbon materials,^[^ ^181^, ^182^, ^183^, ^184^ ^]^ metallic compounds^[^ ^185^, ^186^, ^187^ ^]^ and metal nanoparticles.^[^ ^188^, ^189^, ^190^, ^191^ ^]^ Among these, QDs loaded on conductive carbon hosts are one of the most promising candidates.

### 5.1 Carbon QDs Modified Separators

**Figure 15:**
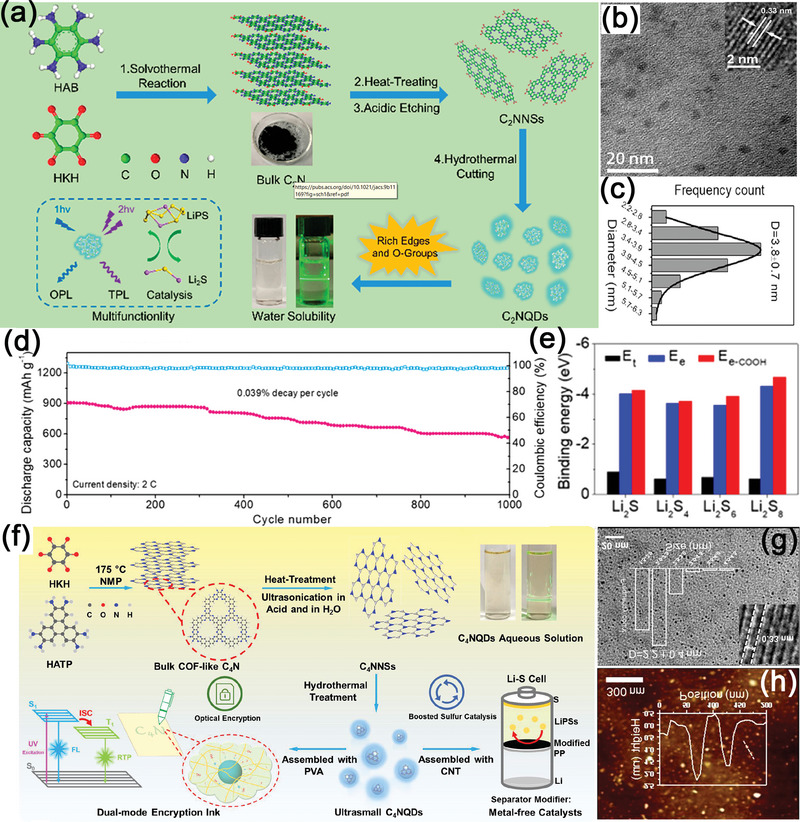
a) The synthesis diagram and observed Tyndall effect of C_2_NQDs in water. b) HRTEM image of C_2_NQDs. c) Statistical analysis of the sizes of C_2_NQDs. d) Binding energies of LiPSs. e) Long‐term cycling stability. Reproduced with permission.^[^ ^191^ ^]^ Copyright 2020, American Association for the Advancement of Science. f) Schematical illustration for the synthesis and practical uses of C_4_NQDs and their composite assemblies. g) TEM image and the size distribution of mono‐dispersed C_4_NQDs. h) AFM image and the corresponding height profiles of C_4_NQDs. Reproduced with permission.^[^ ^195^ ^]^ Copyright 2021, Wiley‐VCH.

As a graphene‐like carbon nitride material with evenly distributed pores, C_2_N has emerged as a new class of appealing 2D frameworks that possess many distinctive advantages, such as large surface area, homogeneous active sites, strong covalent linkages, and a semiconductor band gap (**Figure** **15**a).^[^ ^192^, ^193^ ^]^ In this sense, converting bulk C_2_N into 0D QDs can trigger unique quantum confinement and edge effects to produce improved or new properties. In 2020, Yu et al. demonstrated the first synthesis of water‐soluble C_2_NQDs through an effective “top‐down” approach (Figure 15a).^[^ ^194^ ^]^ The as‐prepared C_2_NQDs with an average size of less than 5 nm were endowed with abundant oxygen‐carrying groups and a high density of exposed active edges (Figure 15b,c). After integrating C_2_NQDs with CNTs and subsequently deposited onto a commercial Celgard separator, the assembled Li–S battery delivered a distinguished cycling stability (fading rate of 0.039% per cycle, after 1000 cycles), and a large area capacity of 7.0 mAh cm^−2^ when S loading increases to 8.0 mg cm^−2^ (Figure 15d). Theoretical calculations (Figure 15e) showed that the binding energies of LiPS interacting with the edge site (E_e_) are markedly larger than those with the terrace site (E_t_), indicating the higher reactivity of N atoms at the C_2_N edge. More importantly, the presence of —COOH groups on C_2_N nanoribbons induced further enhancement in the interaction, highlighting the synergistic role of oxygen groups and active edges in promoting polysulfide immobilization. After that, Yu and co‐workers have prepared an ultra‐small colloidal C_4_N QDs from the predesigned covalent‐organic framework (COF)‐like bulk C_4_N in Figure 15f.^[^ ^195^ ^]^ From the Figure 15g,h, it can be seen that the obtained C_4_N QDs have an average size of 2.2 nm. Surface characterization proved that C_4_N QDs also have high density of edge sites and carbonyl groups, similar to those for C_2_N QDs. Thus, the commercial separators modified by C_4_N QDs@CNT composite also realized a distinguished cycling stability with a decay rate of 0.061% per cycle after 800 cycles at 1.0 C.

**Figure 16:**
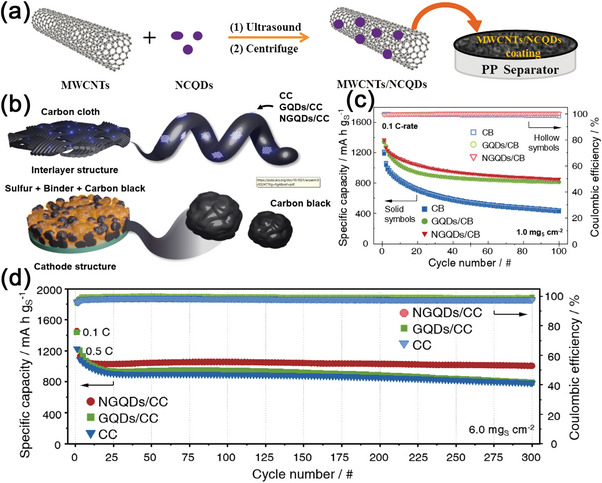
a) Schematic representation of the fabrication of the MWCNTs/NCQDs composite. Reproduced with permission.^[^ ^196^ ^]^ Copyright 2018, Wiley‐VCH. b) Diagram of NGQDs cathode. c) Cycle stability of the NGQDs and other materials. d) Cycle performance over 300 cycles of NGODs on CC electrode. Reproduced with permission.^[^ ^197^ ^]^ Copyright 2021, American Association for the Advancement of Science.

It is critical to control the dosage of composite materials when functionalizing separators for high energy Li–S batteries. As shown in **Figure** **16**a, Xia and co‐workers have reported a novel PP separator, which was decorated by ultralight multiwall carbon nanotubes/N doped CQDs (MWCNTs/NCQDs) composite.^[^ ^196^ ^]^ It is interesting that only a dosage of 0.15 mg cm^−2^ MWCNTs/NCQDs on the separator could enable the production of Li–S cell with a high initial discharge capacity of 1330.8 mAh g^−1^ and a low fading rate of 0.05% per cycle. Besides, the MWCNTs/NCQDs‐coated separator could also effectively depress the self‐discharge issue. This enhanced electrochemical performance could be ascribed to the synergetic effect of MWCNTs and NCQDs, including the improved electrical conductivity, as well as the combination of physical and chemical absorption of polysulfides by the porous MWCNTs network and the oxygenated functional groups/ N atoms on the NCQDs, respectively.

Since CQDs have demonstrated enhanced electrochemical performance either incorporated within sulfur cathodes or decorated on separators, Cairns's group combined them together and reported the functionalization of nitrogen‐functionalized GQDs (NGQDs) both onto carbon blacks (CBs) as a sulfur host in the cathode and onto carbon cloth (CC) as the interlayer (Figure 16b).^[^ ^197^ ^]^ Due to the nitrogen‐rich and oxygen‐containing functional groups on NGQDs, the interlayer and cathode could offer strong sulfiphilic properties through chemical adsorption. As a result, the sulfur cathodes NGQDs/CB coupled with NGQDs/CC separator delivered higher reversibility (Figure 16c) and faster kinetics (Figure 16d) than bare CBs and those decorated with GQDs.

### 5.2 Metal Compounds QDs Modified Separators

**Figure 17:**
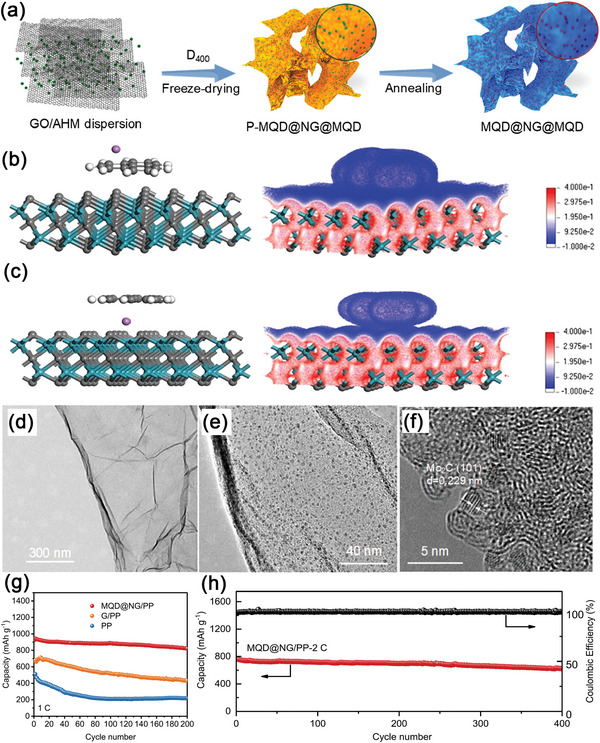
a) Schematic diagrams of the preparation process of MQD@NG. b) The digital photograph of MQD@NG/PP. c‐d) TEM image of MQD@NG. e) Cycling performance of PPG@PP, MQD@NG/PP cells at 1 C. f) Cycling stability of the MQD@NG/PP cell at 2 C. Reproduced with permission.^[^ ^198^ ^]^ Copyright 2020, Elsevier.

Rightful design of metal compound QDs can not only ensure strong chemical polarity to confine the shuttling effect and high conductivity to catalyze the conversation and transformation of the LiPSs, but can also tailor the lithiophiliciy of the QDs for the prevention of lithium dendrite formation. Thus, metal compounds QDs combined with the conductive carbon matrix have been considered as ideal candidates to decorate the commercial PP separator. He and co‐workers have prepared a novel Mo_2_C QDs based bifunctional interlayer, which was fabricated through the adhesion of Mo_2_C QDs onto double sides of N‐doped graphene (NG) nanosheets (MQD@NG/PP) that were further coated onto commercial separators (**Figure** **17**a).^[^ ^198^ ^]^ DFT calculations (Figure 17b) showed that the adsorption energy between MQD@NG and Li^+^ is larger (−3.14 eV) than that between graphene and Li^+^ (−2.60 eV), suggesting that growing Mo_2_C crystal on graphene can enhance the adsorption of Li^+^. Interestingly, the adsorption energy can be further increased to −4.64 eV due to the increased overlap of electron density (Figure 17c). Since ultra‐small Mo_2_C QDs were uniformly anchored on ultra‐thin NG nanosheets (Figure 17d–f), this structure guaranteed the uniform and fast Li^+^ flow onto the surface of Li metal anode, processing homogeneous dendrite‐free Li deposition with ultralong‐term reversible Li plating/stripping. In addition, the highly flexible NG nanosheets could mechanically suppress the implantation of lithium dendrites; the polar Mo_2_C QDs possessed strong and stable chemical adsorption of LiPSs thus effectively alleviate the shuttling effect; the coupling of Mo_2_C QDs and NG nanosheets also ensured high conductivity for fast LiPSs conversion. As a result, in Figure 17g,h, this bifunctional interlayer achieved high capacity as well as ultrastability for Li–S batteries.

**Figure 18:**
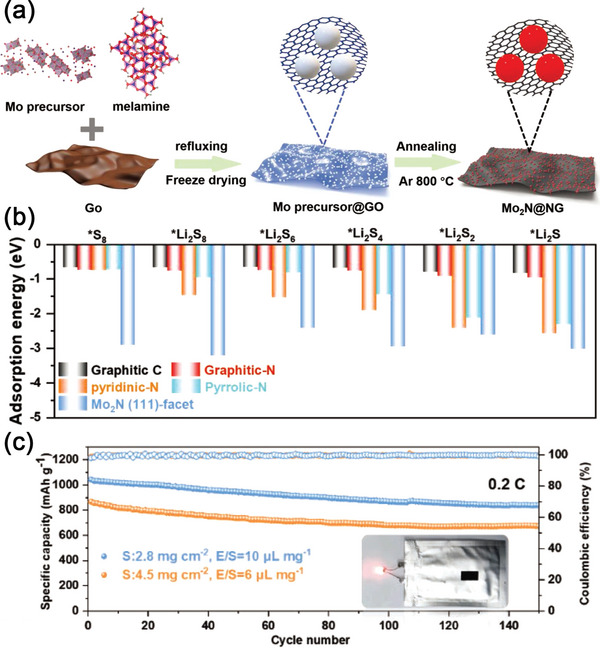
a) Schematic illustration of the synthetic procedure of Mo_2_N@NG. b) Comparison of adsorption energies between LiPSs and Mo_2_N (111) surface. c) Cycling performance of the pouch cell with Mo_2_N@NG/PP separator under various high‐sulfur loadings. Reproduced with permission.^[^ ^199^ ^]^ Copyright 2022, Wiley‐VCH.

Recently, Chen and co‐workers have prepared a novel hybrid material of Mo_2_N QDs anchored onto N‐doped GO (Mo_2_N@NG) as a bifunctional coating on the separator for advanced Li–S batteries, in **Figure** **18**a.^[^ ^199^ ^]^ Theoretical calculations and in situ Raman synergistically elucidated that this hybrid material demonstrated both the sulfiphilic and lithiophilic features which are shown in Figure 18b. Thus, the embedded polar Mo_2_N QDs could significantly suppress the shuttling effect of LiPSs and accelerate the redox reaction between S and Li_2_S; the porous and conductive NG skeleton acted as both 3D electron channels and LiPSs reservoirs; the hybrid framework presented high‐flux Li^+^ diffusion and uniform lithium deposition, successfully mitigating the formation of lithium dendrite growth. Benefiting from these advantages from Mo_2_N QDs@NG modified separators, in Figure 18c the S cathode displayed a high discharge capacity of 964.0 mAh g^−1^ after 300 cycles at 0.5 C under a high S loading of 2.8 mg cm^−2^. More interesting, the symmetric cell kept a stable overpotential (≈4.8 mV for 1000 h and ≈142 mV for 1400 h) and no obvious degradations appeared during Li stripping and plating.

## 6 Conclusions and Perspectives

Li–S batteries have a high theoretical specific capacity and are among the most promising candidates for electro‐mobile and large‐scale energy storage. However, they exhibit various challenges that are associated with sulfur cathodes (poor electrical conductivity, large volume expansion, shuttling effects, and slow reaction kinetics) as well as with Li metal anodes (Li dendrites, large volume change, and repeated formation of SEI film) (Figure 1b). In tackling all these obstacles, QDs, as the novel ultrafine materials (<10 nm) with quantum‐confined structures, have many advantages over conventional micro or nano size of catalytic sites when used in Li–S batteries. Moreover, the QD‐based hybrids are compatible with the current technologies in terms of materials synthesis, electrode preparation, as well as battery fabrication. As such, recent development of QD‐based hybrid materials for Li–S batteries has been reviewed in terms of their synthesis methods and structural designs, from which the reaction mechanisms have been summarized.

**Figure 19:**
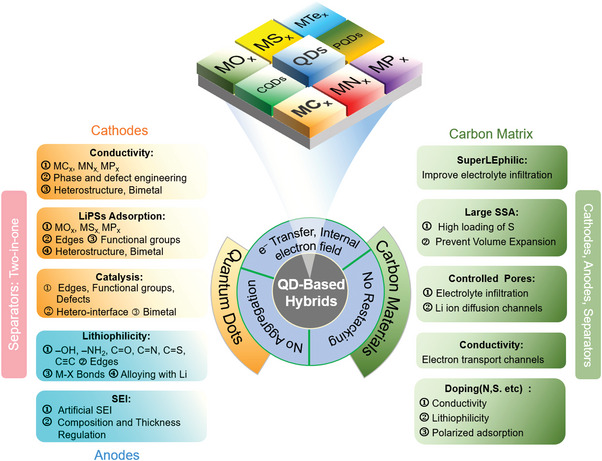
Guidelines of structural designs of QD‐based hybrids with their underlying mechanisms for highly efficient and stable Li–S batteries.

The guidelines of the structural designs of QD‐based hybrid materials with their underlying mechanisms for Li–S batteries have been provided in **Figure** **19**. Highly‐porous and conductive carbon hosts decorated by QDs can increase the sulfur loading, facilitate the electron/Li^+^ transport, and buffer the volume expansion, because of the prevention of the aggregation of QDs and the restacking of carbon hosts, as well as the establishment of internal electron field by electron transfer between QDs and carbon hosts. On sulfur cathodes, QDs can also improve the electrical conductivity of whole electrodes and effectively restrict the shuttling effects of LiPSs, due to the facile modulation of their electronic structure and chemical polarity. In addition, QDs can promote the reaction kinetics by catalyzing the conversion reactions and accelerating the solid‐state migration of LiPSs to the active sites, due to their quantum‐confined structures with diversified strategies in improving the reaction activity. On lithium metal anodes, QD‐based hybrid materials can regulate Li deposition and prevent the dendrite formation, owing to the synergistic effects of increasing lithiophilicity and the exposure of the lithiophilic sites. For modified separators, QD‐based hybrid materials can integrate advantages demonstrated in cathodes and anodes, and offer extra protection on the way of the shuttling of polysulfide anions and Li^+^ cations. All these features satisfy the demands for advanced Li–S batteries, endowing QDs with great potential to be used as irreplaceable additives for the electrodes, electrolytes, and interlayers.

Even though the applications of QDs in advanced Li–S batteries have made major progress, the difficulties of using QD‐based hybrids, their further development and commercial applications in the field are discussed further, associated with suggested solutions and perspectives.

1. Many studies have reported outstanding electrochemical performance after incorporating QDs in Li–S batteries; however, it is still far away from commercial applications since the mass production of QDs or QD‐based hybrid materials is still doubtful. There is an urgent need to develop a simple and scalable method to realize large production of QDs with a consistent performance in Li–S batteries.
2. Although QDs have unique morphological and electronic features for the catalytic reactions in Li–S batteries, their structural evolution during the charge/discharge process has not been fully established. As the main reasons of deactivation, QDs with high surface energy have tendency to agglomerate into large particles, which would lead to the disappearance of the quantum confinement effects. In addition, the structural changes of the active sites during cycling and the coverage of the active sites by the products from the side‐reactions at the interface are also the causes of deactivation. Thus, the relationship between structural changes and capacity fading should be investigated further to improve the performance of QD‐based electrodes. Although the roles of QDs in Li–S batteries have been elucidated to some extent via experimental and theoretical calculations, previous studies lacked real‐time, atomic‐scale insights on the conversion of LiPSs on QDs, and the changes of electronic structures of QDs during the reactions of Li–S batteries. Therefore, novel in situ microscopic/spectroscopic analyses (such as in situ electrochemical TEM and in situ synchrotron) or other advanced characterization methods should be developed and performed for in‐depth investigations of reaction mechanisms.
3. Studies have reported that QDs with a large surface area are beneficial for energy storage. However, they could also lead to the generation of a thick SEI and unwanted side reactions, consuming a large volume of electrolytes. During this deactivation process, the electrolytes may drain out and cause capacity fading. Unfortunately, the requirement of a controlled amount of lean electrolyte within Li–S batteries has been neglected in most studies. Future studies should elucidate the relationship between the electrolyte dose and the electrochemical performance after adding QD‐based hybrid additives in Li–S batteries.
4. To obtain high electrical conductivity, QDs are normally combined with carbon matrix as the host material for sulfur cathodes or for Li metal anodes. However, the non‐polar and lithiophobic nature of carbon hosts can lead to poor adsorption ability toward LiPSs and non‐uniform lithium deposition. The polar and lithiophilic properties can be improved via heteroatom doping (N, S, etc.). Nonetheless, undesirable side reactions may also occur between the heteroatoms and act materials (sulfur species, lithium ion), which could worsen the system stability leading to capacity fading. Therefore, it is critical to develop new types of conductive matrices in carrying QDs.
5. Most current studies of QDs in Li–S batteries are based on monometallic compounds and few on bimetallic systems. When more than two metallic carbides, nitrides, oxides, and phosphides are introduced into electrode materials, multiple redox centers will be present, which may generate synergistic effects in improving the catalytic activities and enhancing the migration of LiPSs on the solid surface of the catalysts. Future studies are suggested in systematically evaluating different multi‐metallic system based QDs as well as their synergistic mechanisms. Catalyst library may be built, from which the rightful QD‐based materials can be selected for tackling specific problems in Li–S batteries.

In conclusion, given the excellent conductivity, large surface area, strong adsorption capacities toward LiPSs, excellent catalytic performance, and plenty of lithiophilic nucleation centers, QD‐based hybrid materials have demonstrated great potential to overcome the limitations associated with sulfur cathodes as well as lithium metal anodes, which is believed to promote the commercialization of Li–S batteries in the future.

## Conflict of Interest

The authors declare no conflict of interest.
